# Uridine-cytidine kinase 2 is correlated with immune, DNA damage repair and promotion of cancer stemness in pan-cancer

**DOI:** 10.3389/fonc.2025.1503300

**Published:** 2025-01-27

**Authors:** Jinlong Tian, Yanlei Li, Yu Tong, Yuan Zhang, Tingxiao Zhao, Yao Kang, Qing Bi

**Affiliations:** ^1^ Graduate School of Bengbu Medical University, Bengbu, Anhui, China; ^2^ Sports Medicine Center, Department of Orthopedics, Zhejiang Provincial People’s Hospital (Affiliated People’s Hospital, Hangzhou Medical College), Hangzhou, China; ^3^ Department of Rheumatology and Immunology, The Second Affiliated Hospital of Zhejiang University of Traditional Chinese Medicine, Hangzhou, China

**Keywords:** pan-cancer, uridine-cytidine kinase 2, DNA mismatch repair (MMR), homologous recombination repair (HRR), cancer stemness, immune, major histocompatibility complex (MHC), drug sensitivity

## Abstract

**Background:**

UCK2 (Uridine-Cytidine Kinase 2) is a promising prognostic marker for malignant tumors, but its association with immune infiltration and cancer stemness in pan-cancer remains to be fully understood. we find that gene UCK2 is closed related to RNA stemness scores (RNAss) and DNA stemness scores (DNAss), which is measured the tumor stemness. We also discover an association between UCK2 expression and immune cells by CIBERSORT algorithm, ESTIMATE algorithm and ssGSEA algorithm, especially, related to T cell, monocytes, mast cells, and macrophages. This study aims to shed light on the role and possible mechanism of UCK2 in pan-cancer.

**Methods:**

We used the R programming language for pan-cancer bulk sequencing data analysis, which were obtained from the University of California, Santa Cruz (UCSC) datasets. UCSC database is a very useful for explore data from TCGA and other cancer genomics datasets, The data we explored at the UCK2 transcriptome level came from TCGA data in the UCSC database. We explored differential UCK2 expression between tumor and normal samples. Immunohistochemistry (IHC) was utilized to validate the expression of UCK2 in different types cancers using tumor tissue chips. The correlations of UCK2 with prognosis, genetic instability, DNA repair, cancer stem cell characteristics, and immune cell infiltration were investigated. Furthermore, single-cell datasets, acquired from the Gene Expression Omnibus (GEO) database, were used to validate the relationship between UCK2 and immune cells. GEO is a famous public genomics database supporting freely disseminates microarray data. Finally, we analyzed the correlation between UCK2 and drug sensitivity.

**Results:**

UCK2 expression was observed to be high in most cancers and was remarkably related to the prognosis of pan-cancers. We found that the increased UCK2 expression was associated with higher genetic instability. Additionally, positive relationships were observed between UCK2 expression and mismatch repair genes, homologous recombination repair genes, and cancer stemness across different cancer types. There were significant correlations between UCK2 and T cells, monocytes, mast cells, and macrophages. Moreover, as expected, the immune checkpoint human leucocyte antigen (HLA) was found to be negatively related to UCK2. Similarly, UCK2 was also observed to have a negative association with major histocompatibility complex (MHC) genes. We noted that UCK2 had significant correlations with the sensitivity to various anti-cancer drug.

**Conclusion:**

We have observed that UCK2 plays pivotal roles in prognosis and tumor immunity, and it is associated with DNA repair and cancer stemness. The UCK2 gene exhibits a strong correlation with the immune checkpoints HLA. This study highlights its potential impact on drug sensitivity.

## Introduction

Cancer is a difficult-to-understand disease with serious consequences, causing great suffering to patients and increasing the social and economic burden. In 2024, there will be 2001140 cancer deaths in the United States, and 611,720 cancer-related deaths. Between 2015 and 2019, the prevalence of pancreatic cancer and liver cancer (women) increased by 0.6% to 1% and 2% to 3%, respectively. The cancers that currently have the lowest survival rates include pancreatic, liver, esophageal and lung cancers. As a result of immunotherapy and targeted therapies, as well as screening for early-stage cancers, cancer mortality declined by 33 percent from 1991 to 2021 (1). Therefore, the discovery of new prognostic biomarkers is very important. Before 1970, treatments for cancer included surgery, radiation therapy, chemotherapy and Allogeneic hematopoietic stem cell transplantation, Between 1970 and 2023, the number of procedures for cancer treatment increased by six including Pharmacological hormone therapy, Treatments targeting genes with oncogenic alterations and treatments related signaling pathways, Photodynamic therapy, Antibody drug conjugates, Immune check point inhibitors, Bispecific T-cell engagers, Oncolytic virus therapy, Chimeric antigen receptor T cell therapy, although there are so many treatments, there are still many cancer patients (2). Following cardiovascular disease, cancer becomes the second main cause of mortality in the United States of America, As the descending mortality of cardiovascular disease, cancer becomes the first cause of death in a part of countries (3, 4), To better know the mechanism of cancers and effectively prevent it, there is a focus on key gene that drive the development of cancer. Regrettably, the study of far-reaching gene in single cancer research is not only limited to make its results applied to other cancers, but also difficult to comprehensive understand the evolution of cancer. Pan-cancer analysis is of great clinical significance, which can find common biomarkers of multiple cancers, and can also predict the diagnosis, treatment and prognosis of multiple cancers, Recently, many researchers have done this kind of research and achieved success (5–11), pan-cancer research can also understand the general mechanism of cancer occurrence, and recently, researchers have revealed the general cancer mechanism through the development of biomarkers and clinical relevance analysis (12, 13). Hence, the pursuit of pan-cancer investigation of genes has arisen as a pragmatic strategy for unraveling the enigma of cancer gene, while current pan-cancer studies have provided limited insight due to the absence of integrative multi-omics or polysome profile analyses.

Humans possess two UCK genes, namely UCK1 (Uridine-Cytidine Kinase 1) and UCK2 (Uridine-Cytidine Kinase 2) (14). Nucleotides are the basis of all cellular metabolic processes, but an imbalance of these nucleotides can lead to interference with genetic damage (15), Uridine cytidine kinase (UCK) is a rate-limiting enzyme in the rescue pathway of pyrimidine nucleotide synthesis, Pyrimidine metabolism is essential for DNA replication RNA synthesis and cellular bioenergy and for cancer cells to maintain uncontrolled tumor growth through a continuous supply of dNTP (16) UCK2 phosphorylates uridine and cytidine to uridine monophosphate (UMP) and cytidine monophosphate (CMP), respectively (17), in the presence of uridine cytidine kinase (UCK), uridine and cytidine nucleoside are required to facilitate transport and subsequent phosphorylation to synthesize RNA and DNA (18). They both facilitate the conversion of uridine and cytidine to their monophosphate form, which is crucial for the production of pyrimidine nucleotides used in DNA and RNA synthesis (14, 18–21). Despite their similar catalytic functions, UCK2 exhibits 15-20 times greater catalytic potency in uridine and cytosine compared to UCK1 (14). The expression of UCK2 is elevated in various cancer tissues, including breast cancer, hepatocellular carcinoma, lung cancer, it is considered a prognostic biomarker for these cancers (22–24). Despite the reported involvement of UCK2 in certain cancer types, there remains a significant dearth of research regarding its impact across the entire spectrum of cancer. As the same time, an article suggests that the gene UCK2 should be used for pan-cancer research (25).

The DNA damage repair process enables cells to repair DNA damage resulting from various factors, including aging, exposure to substances, viral infections, and natural radiation. Homologous recombination repair is considered the most crucial and accurate form of DNA repair in numerous repair systems (26). When a double-strand DNA break occurs, the Homologous recombination repair pathway is triggered to facilitate its repair. On the other hand, the correction of DNA replication errors is a critical function of DNA mismatch repair (MMR). Cancers employ MMR and HRR mechanisms to sustain genome stability, stemness, chemoresistance (27, 28). A prior study on lung cancer has indicated a correlation between UCK2 and DNA repair (24). In this study, we aim to investigate the association between UCK2 and DNA repair across pan-cancer.

The tumor microenvironment (TME) is a highly intricate and dynamically evolving system, resulting from the complex interplay between tumor cells, immune cells, stromal cells, and extracellular matrix components (29, 30). The TME play a crucial role in the pathogenesis, progression, and therapeutic outcomes of various tumors, demonstrating both stimulatory and inhibitory effects on tumor growth, invasion, and metastasis (30–32). In this context, the contribution of memory CD4+ T cells to these processes is unequivocal (33, 34). Cluster of differentiation 4 (CD4) memory T cells are antigen-specific effector memory CD4+ T cell subsets that are retained following the expansion, contraction, and memory phases of primary T cell response (35, 36). CD4+ T cell has been associated with unfavorable outcomes in various malignancies, including renal cell carcinoma, prostate adenocarcinoma, non-small cell lung cancer, and breast carcinoma (37–40). In recent years, research on tumor immunotherapy has experienced rapid development. However immune therapy faces some challenge, such as adverse events, high expensive cost and side effect (41). The new method of immune therapy needs to be urgently explored. UCK2 is an enzyme involved in pyrimidine nucleotide metabolism, and its increased activity has been linked to the occurrence and progression of several types of cancer, including breast, lung, and colon cancer (22, 24, 42), Due to the abnormal expression of UCK2 in these cancers, targeting UCK2 has been identified as a potential avenue for cancer treatment. The rationale for the use of UCK2 as a target for cancer therapy is that it is expressed at a high level in tumor cells and relatively low in normal tissues. This makes it possible to specifically inhibit UCK2 enzyme activity, theoretically reducing the damage to normal cells and improving the safety of treatment. Inhibition of the UCK2 enzyme may interfere with DNA synthesis in cancer cells, thereby inhibiting the growth and proliferation of cancer cells. By targeting UCK2, the progression of hepatocellular carcinoma can be inhibited, potentially leading to improved response to immunotherapy in patients with this disease (43). At present, there are still some gaps in the research on the role of UCK2 in immune interaction and stemness in different cancers, UCK2, as an enzyme involved in pyrimidine nucleotide metabolism (17), has been gradually recognized for its role in tumor development, but its interaction with the immune system and the specific mechanism of action in different types of cancer have not yet been fully revealed. Recently, only in liver cancer, the interaction between gene UCK2 and immunity has been studied more, and other cancers have been involved less (43, 44). For the study of cancer cell stemness, the gene UCK2 was mentioned in a nine-gene-based stemness classifier prediction model designed to predict immunotherapy response and prognosis of liver cancer (44). Therefore, the role of UCK2 in immune interaction and coordination in other cancers deserves further exploration.

This study has conducted a thorough analysis of UCK2 profiles, including its clinical features, single-cell sequencing, and particularly its roles in DNA repair and cancer immunity. Furthermore, our findings provide a comprehensive understanding of the roles of UCK2 in cancers and offer valuable insights for the development of novel targeted therapies.

## Materials and methods

### Data collection and procession

The batch-corrected and normalized pan-cancer and normal tissue datasets were acquired from the University of California, Santa Cruz (UCSC, xena. http://xena.ucsc.edu/, accessed on 2 December 2022) datasets, which included The Cancer Genome Atlas (TCGA, encompassing 33 cancer types), Therapeutically Applicable Research to Generate Effective Treatments (TARGET, consisting of 7 pediatric cancers), and Genotype Tissue Expression (GTEx, comprising of 54 normal tissues). Kidney Chromophobe (KICH), Kidney Clear Cell Carcinoma (KIRC), Kidney Papillary Cell Carcinoma (KIRP), Glioblastoma Multiforme (GBM), Brain Lower Grade Glioma (LGG), Lung Adenocarcinoma (LUAD), and Lung Squamous Cell Carcinoma (LUSC) have a smaller number of corresponding normal samples available in the TCGA dataset. Consequently, these samples were combined with paired normal samples from the GTEx dataset. However, for other types of cancers, there were no matching normal samples found in GTEx, despite the absence of normal samples in the TCGA dataset. Specifically, expression data for cell lines was collected from the Cancer Cell Line Encyclopedia (CCLE) (45). The dataset for TCGA and GTEx was downloaded from the UCSC xena website, the dataset we downloaded was TCGA TARGET GTEx (13 datasets), the data were normalized using TPM, log2 (x+0.001) was transformed, and meta-analysis was used to make the TCGA data better compared to GTEx (https://ucsc-xena.gitbook.io/project/faq/advanced-data-and-datasets).

We retrieved single-cell sequencing datasets for bladder urothelial carcinoma (BLCA) (GSE145137), cholangiocarcinoma (CHOL) (GSE125449), head and neck squamous cell carcinoma (HNSCC) (GSE103322), kidney renal clear cell carcinoma (KIRC) (GSE171306), liver hepatocellular carcinoma (LIHC) (GSE125449), bile duct cancer (CHOL) (GSE125449), ovarian serous cystadenocarcinoma (OV) (GSE118828), prostate adenocarcinoma (PRAD) (GSE137829), stomach adenocarcinoma (STAD) (GSE183904), breast cancer (BRCA) (GSE138536), and melanoma (SKCM) GSE72056 from the Gene Expression Omnibus (GEO) database (https://www.ncbi.nlm.nih.gov/geo/). To address sample-to-sample variation in single-cell data from multiple samples, we employed the R-package “harmony” (46) for integration. This approach allowed us to effectively integrate and harmonize the datasets, enabling us to obtain more accurate and reliable results. We employed the Uniform Manifold Approximation and Projection (UMAP) function for dimensionality reduction in our visualization. This approach allowed us to effectively reduce the complexity of the data and visualize it in a more simplified and informative manner. Additionally, the Cellmarker website (47) was utilized to further enhance the accuracy of cell annotation.

### Differences in UCK2 expression between normal, cancerous, and various tissue stages

The differential expression of UCK2 in cancer and normal tissues was analyzed and visualized using the “ggplot2” package in R software version 4.2.3, which is an open-source program available on The Comprehensive R Archive Network as of March 15, 2023. To assess the differential expression of UCK2 across different cancer stages, two sets of Wilcoxon tests were conducted. Meanwhile, we have compared the differential expression of cancers and adjacent tissues in TCGA datasets. Additionally, we have utilized the GPSAdb database (http://guotosky.vip:13838/gpsa/) and R programming language to investigate the expression of UCK2 in the TCGA, GTEx, and CCLE datasets.

### Tissue microarray and IHC analyses

Pan-carcinogenic tissue chips containing 20 types of tumors were purchased from Shanghai Outdo Biotech Company. Two tumor chips (non-small cell lung cancer and melanoma) were excluded because they only contained two samples. The experimental steps of immunohistochemistry mainly include: Firstly, paraffin-embedded tissue sections undergo the process of deparaffinization by heating them in an oven at 63 degrees for one hour, followed by treatment with xylene and hydration through a gradient of ethanol solutions. Secondly, the primary antibody (UCK2, 1:1500 dilution, 10511-1-AP, proteintech) was used and allowed to incubate overnight at 4°C within a controlled humidified environment. Subsequently, the corresponding secondary antibody was administered. The slides are then placed into a DAKO automatic immunohistochemistry instrument and the corresponding program is run for blocking, secondary antibody binding, and DAB color development procedures following the “Autostainer Link 48 User Guide”. Lastly, hematoxylin counterstaining and neutral resin mounting are carried out. All experimental procedures strictly adhered to the instructions provided with the kit. The staining intensity of each sample is represented by the average gray value. we performed a quantitative analysis using Fiji (1.54f) software by capturing two random microscopic images of each section. At least 3 replicates per sample. We used the average optical density (The Integrated Optical Density value divided by the area of the target distribution area) value to quantify the staining intensity. We numbered the slices according to the principle of randomization. Two experienced pathologists were invited to read the slices in a single-blind way, and summarized and analyzed according to the numbers of random groups.

### Survival analysis

The prognostic value of UCK2 was analyzed using univariate Cox regression analysis for overall survival (OS), disease-free interval (DFI), disease-specific survival (DSS), and progression-free interval (PFI). This analysis was carried out in 33 different cancer types and presented visually in a “forestplot” (version 3.1.1). To determine the optimal cutoff value for dividing UCK2 expression into two groups, we utilized the “sur_cutpoint” function from the “survminer” package. The sur_cutpoint function uses the ‘maxstat’ R package to select the maximum rank statistic, determines the best cut-off point for one or more continuous variables at a time, and finally selects the potential cut-off value with the largest difference between groups as the final cut-off value. The “survfit” and “ggsurvplot” functions were then used to analyze and visualize the survival differences through Kaplan-Meier curves.

### Genetic analysis of UCK2 alteration

We utilized the cBioPortal database (48) to analyze the relationship between UCK2 and copy number variation and mutation. Additionally, Pearson’s correlation analysis was conducted to determine the association between copy number variations (CNVs), DNA methylation levels, and UCK2 expression. To analyze the association between Promoter DNA methylation and UCK2, we used the UALCAN dataset (49, 50). The Tumor Mutation Burden (TMB) was determined using the “maftools” package, and the dataset on Masked Somatic Mutation from TCGA was downloaded for this purpose. Furthermore, the Homologous Recombination Deficiency (HRD) data was obtained from a study (51), while the Microsatellite Instability (MSI) data was collected from the “Bioc0ncoTK” package. Finally, this study presented the correlation between HRD, TMB, MSI, and the expression of UCK2.

### Investigating correlation of UCK2 with DNA mismatch repair, cancer stem and epigenetic modification

The data on DNA methylation-based stemness scores (DNAss) and mRNA expression-based stemness scores (RNAss) were collected from the UCSC database to calculate their correlation with UCK2. The association between 5 mismatch repair genes (MMR) (52), 4 DNA methyltransferase genes (53), Homologous Recombination Repair (HRR)-related gene (54) and UCK2 expression in various cancers was visualized.

### Functional enrichment analysis

The gene list comprising the most relevant genes correlated with UCK2 were uploaded to the Database for Annotation, Visualization, and Integrated Discovery (DAVID, v2022q4) (https://david.ncifcrf.gov/tools.jsp), for further analysis and annotation. Then, to get enrichment results, it is recommended to select the official gene symbol in many identifiers and choose Homo sapiens in all kinds of species. Finally, the enrichment results of Gene Ontology (GO) analysis and Kyoto Encyclopedia of Genes and Genomes (KEGG) pathway analysis were obtained. To gain a further understanding of UCK2 biological processes, gene set enrichment analysis (GSEA) was also conducted using hallmark gene sets downloaded from the MSigDB database.

### The role of UCK2 expression in tumor immune microenvironment

The study analyzed the impact of UCK2 on microenvironment infiltration in various types of cancer by utilizing the Estimation of Stromal and Immune cells in MAlignant Tumor tissues using Expression data (ESTIMATE) algorithm in the R package “ESTIMATE” (version 1.0.13) (55), including the assessment of ESTIMATE, stromal and immune scores. The immune checkpoint markers data collected from a previous study (56) were utilized to study the correlation of UCK2 expression, the immune subtypes data was acquired from the UCSC, and any immune subtypes containing less than three samples were excluded from the investigation into the relevance of UCK2 expression. The comparison between these subtypes was conducted in pan-cancer using the “ggstatsplot” (vision 0.11.0) package in the R programming language. TISIDB, a comprehensive repository portal dedicated to the study of tumor-immune system interactions, provides vital data on major histocompatibility complex (MHC) genes, immune stimulator and inhibitor genes, chemokine genes, and chemokine receptor genes. In the R programming language, by applying Spearman’s rank correlation test and the modified “pheatmap” package, a heatmap was generated to demonstrate the associations between these genes and UCK2 expression. The immunocyte infiltrating correlations of UCK2 were subsequently calculated using the CIBERSORT algorithm (57). The immune cell markers, obtained from a previous study (58), were explored and visualized the correlation of UCK2 expression by the packages “Hmisc”, “GSVA” and “ggplot2” in the R programming language.

### Analysis of single cell data

The quality control of our single-cell data analysis is as follows: filter out cells with more than 20% mitochondrial gene expression, which helps to remove cells that may be abnormally expressing mitochondrial genes due to cellular stress or damage, and filter out cells with more than 2500 expression signatures, which may be to remove high-expression cells, which may represent a technical or biological abnormality, such as certain stages of the cell cycle. Cells with less than 200 expression features are filtered out, which helps remove cells with insufficient sequencing depth or high background noise. When multiple samples are involved, we use R package harmony to remove batch effects (46).

### Drug sensitivity analysis

We have gathered data on the IC50 values and the corresponding gene expression of mRNA from two database - the Genomics of Drug Sensitivity in Cancer database (GDSC) and Cancer Therapeutics Response Portal (CTRP). This data has been integrated into oncopredict, a website (http://osf.io/c6tfx/) (59). Though our analysis, we have investigated the correlation between IC50 and UCK2 expression. Additionally, we have collected drug sensitivity and gene expression data from the CellMiner dataset (60) and used the R programing language to analyze the correlation between UCK2 expression and drug sensitivity.

### Statistical analysis

Unless otherwise specified, all bioinformatics analysis were conducted using the R programming language. The survival disparity between the groups was illustrated using Kaplan-Meier curves. Pearson’s or Spearman’s test was utilized to determine the strength of the correlations between variables, depending on the appropriate method. When applicable, either the t-test or Wilcoxon test was utilized to analyze of the disparity between the two samples. R software (version 4.2.3 https://www.R-project.org, 15 march 2023) were used for statistical analysis and visualization. A p-value below 0.05 indicates statistical significance.

## Results

### UCK2 expression in pan-cancer

**Figure 1:**
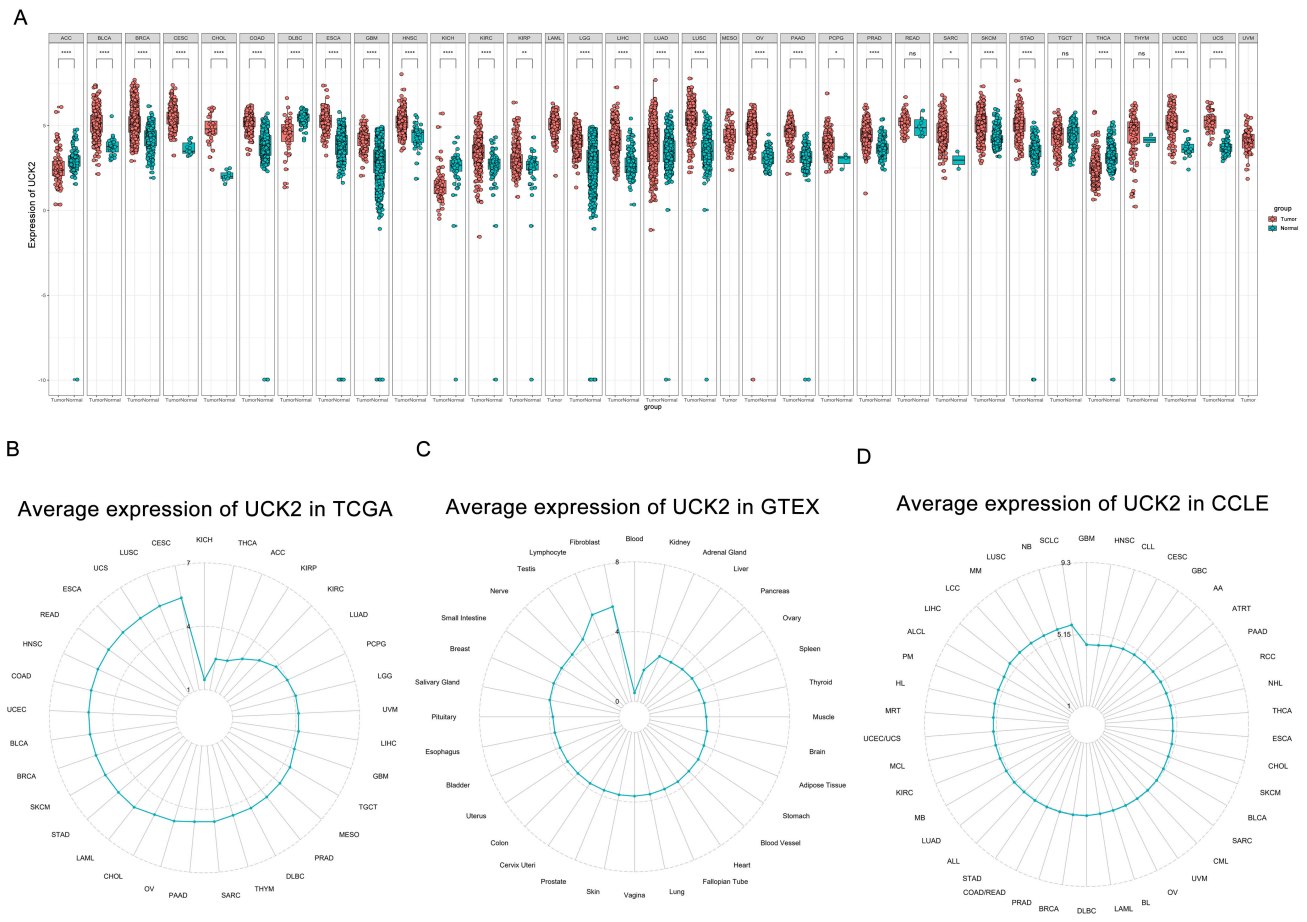
Differential expression of UCK2 observed across various types of human cancers. **(A)** Comparison of UCK2 expression in tumor samples versus normal samples, using data from TCGA and GTEx. **(B)** Expression of UCK2 in 33 types of cancers, according to data from the TCGA database. **(C)** Expression of UCK2 in normal tissues, using data from the GTEx dataset. **(D)** Expression of UCK2 in tumor cells. Using data from the CCLE database. *p < 0.05, **p < 0.01, ****p < 0.0001, ns, not significant.

The gene UCK2 demonstrated significant expression levels in 27 type of cancer samples, it exhibits high expression levels across in 23 cancer types and low expression levels across in 4 cancer types (**Figure 1A**). We conducted an analysis of UCK2 expression using TCGA datasets with normal samples removed, as well as GTEx datasets containing normal samples, CCLE datasets containing tumor samples. In the pan-cancer analysis, UCK2 exhibited the highest level of enrichment in CESC and the lowest level of enrichment in KICH (**Figure 1B**). As presented in **Figure 1C**, UCK2 expression was relatively higher in various issues, including fibroblasts, lymphocytes, testes, nerves and small intestine. UCK2 may be an important target for reversing heart failure. Cardiac fibroblasts are the most important cells mediating the occurrence of myocardial fibrosis, UCK2 is highly expressed in fibroblasts, and inhibition of UCK2 expression may slow down or even reverse heart failure. Recent studies have also shown that UCK2 is an important gene related to heart failure (61). UCK2 is highly expressed in lymphocytes and may play an important role in the immune system, promoting proliferation in the T cell immune response (62) for effective response to infection or tumor. Furthermore, analysis of the CCLE dataset revealed that UCK2 was expressed in all tumor cell lines (**Figure 1D**). Moreover, the GPSAdb database (https://www.gpsadb.com/) (63) verified that UCK2 mRNA expression were consistent (**Supplementary Figures S1A–C**). The expression of UCK2 is significantly associated with the pathologic stage in five types of cancer: NHSC, KIRP, LUAD, LUSC, TGCT (**Supplementary Figures S2A–E**). In addition, we compared the expression levels of UCK2 in tumor and paired adjacent normal tissues using TCGA datasets. Our analysis revealed that UCK2 was highly expressed in several cancer types, including BLCA, BRCA, COAD, HNSC, KIRC, KIRP, LIHC, LUAD, LUSC, PRAD, and STAD, while it was found to be lowly expressed in KICH (**Supplementary Figure S1D**).

### Immunohistochemistry

**Figure 2:**
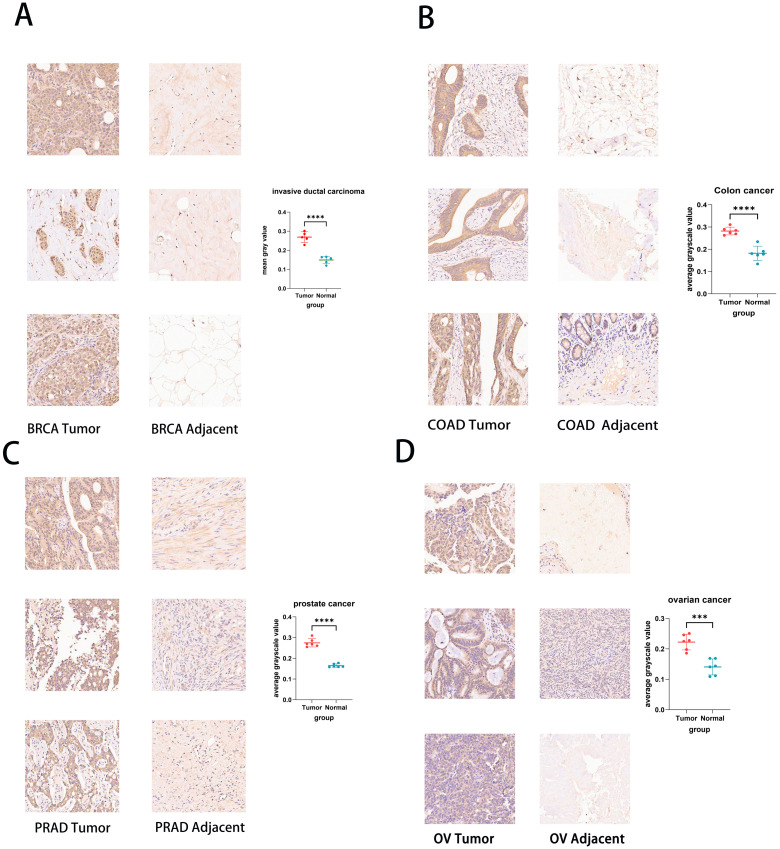
Immunohistochemistry of UCK2 in pan-cancer tissue chips and Statistical analysis of staining intensity. **(A)** BRCA, **(B)** COAD, **(C)** PRAD, **(D)** OV. ***p < 0.001, ****p < 0.0001.

We use the experimental method of IHC to further test and verify the expression of UCK2 in various cancers. We found that the expression of UCK2 was highly expressed in cancer tissue disease compared to normal tissue, for example, BRCA, COAD, PRAD, OV, BLCA, Endometrial cancer (EC), PAAD, READ, STAD, LUAD, and Cervical squamous cell carcinoma (CSCC) (**Figure 2**; **Supplementary Figures S3**, **S4**). These findings mostly align with the results obtained from our bioinformatics analysis. Regarding the expression of UCK2 in EC and CSCC, we did not perform bioinformatics analysis. Instead, compared with cancer tissues, the UCK2 gene is highly expressed in normal tissues, including GBM, KIRC, LIHC, THCA, and Lymphadenoma (**Supplementary Figure S5**).

### The prognosis of UCK2 in pan-cancer

**Figure 3:**
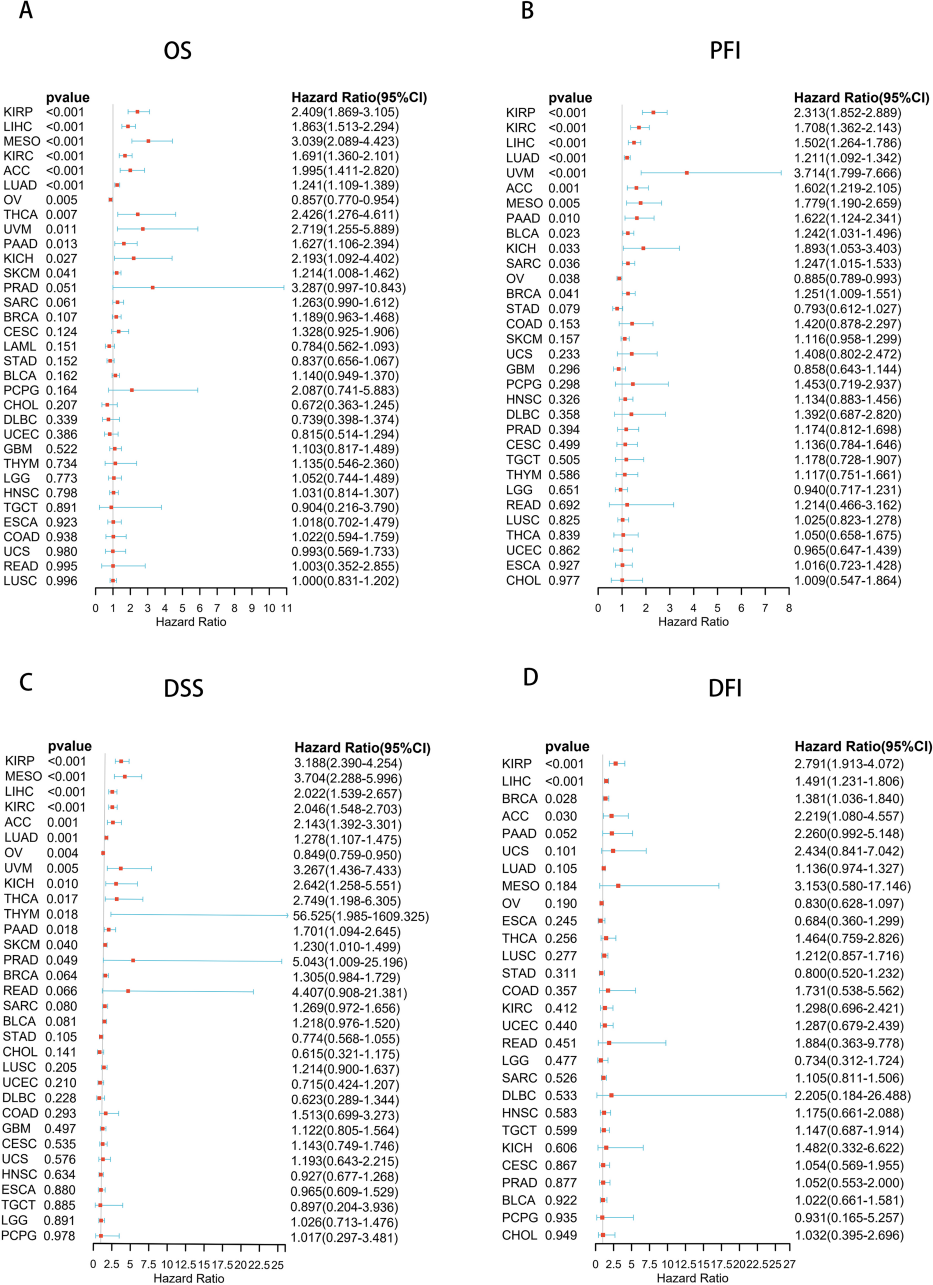
Forest plots based on univariate Cox regression analyse, showing the relationship between UCK2 mRNA expression and **(A)** overall survival (OS), **(B)** progression-free interval (PFI), **(C)** disease-specific survival (DSS), and **(D)** disease-free interval (DFI).

To explore the prognostic value of UCK2, we analyzed the prognostic risk of UCK2 using COX regression analysis. This study examined the relationship between UCK2 mRNA expression and long-term survival across various cancer types. Our analysis revealed a strong correlation between UCK2 expression and overall survival (OS) in twelve distinct cancers, including KIRP (hazard ratio (HR) = 2.409, 95% confidence interval (CI): 1.869–3.105, p < 0.001), LIHC (HR = 1.863, 95% (CI): 1.513–2.294, p < 0.001), MESO (HR = 3.039, 95% (CI): 2.089–4.423, p < 0.001), KIRC (HR = 1.691, 95% (CI): 1.360–2.101, p < 0.001), ACC (HR = 1.995, 95% (CI): 1.411–2.820, p < 0.001), LUAD (HR = 1.241, 95% (CI): 1.109–1.389, p < 0.001), OV (HR = 0.857,95% (CI): 0.770–0.954, p = 0.005), THCA (HR = 2.426, 95% (CI): 1.276–4.611, p = 0.007), UVM (HR = 2.719, 95% (CI): 1.255–5.889, p = 0.011), PAAD (HR = 1.627, 95% (CI): 1.106–2.394, p = 0.013), KICH (HR = 2.193, 95% (CI): 1.092–4.402, p = 0.027), and SKCM (HR = 1.214, 95% (CI): 1.008–1.462, p = 0.041) (**Figure 3A**; **Supplementary Figure S6**). Additionally, the expression of UCK2 showed a significant correlation with PFI (**Figure 3B**), DSS (**Figure 3C**), and DFI (**Figure 3D**). Recently, many studies have shown that in hepatocellular carcinoma, the gene UCK2 is closely related to the immune microenvironment (43, 64), and it can promote normal and malignant T cell proliferation (62), UCK2 is closely related to LIHC survival, we believe that it may affect the tumor immune microenvironment and inhibit the activity of T cells, thus affecting the overall survival rate of patients. UCK2 is a metabolism-related gene (65), it may increase cell proliferation and inhibit cell apoptosis by promoting metabolism-related pathway activation, thereby leading to shortened survival. Studies have shown that UCK2 is related to induction of tumor cell apoptosis (66), perhaps the apoptosis of MESO and ACC cancer cells is related to the involvement of UCK2, which affects the length of survival through the apoptosis pathway.

### Analysis of genetic alterations and instability, methylation in UCK2

**Figure 4:**
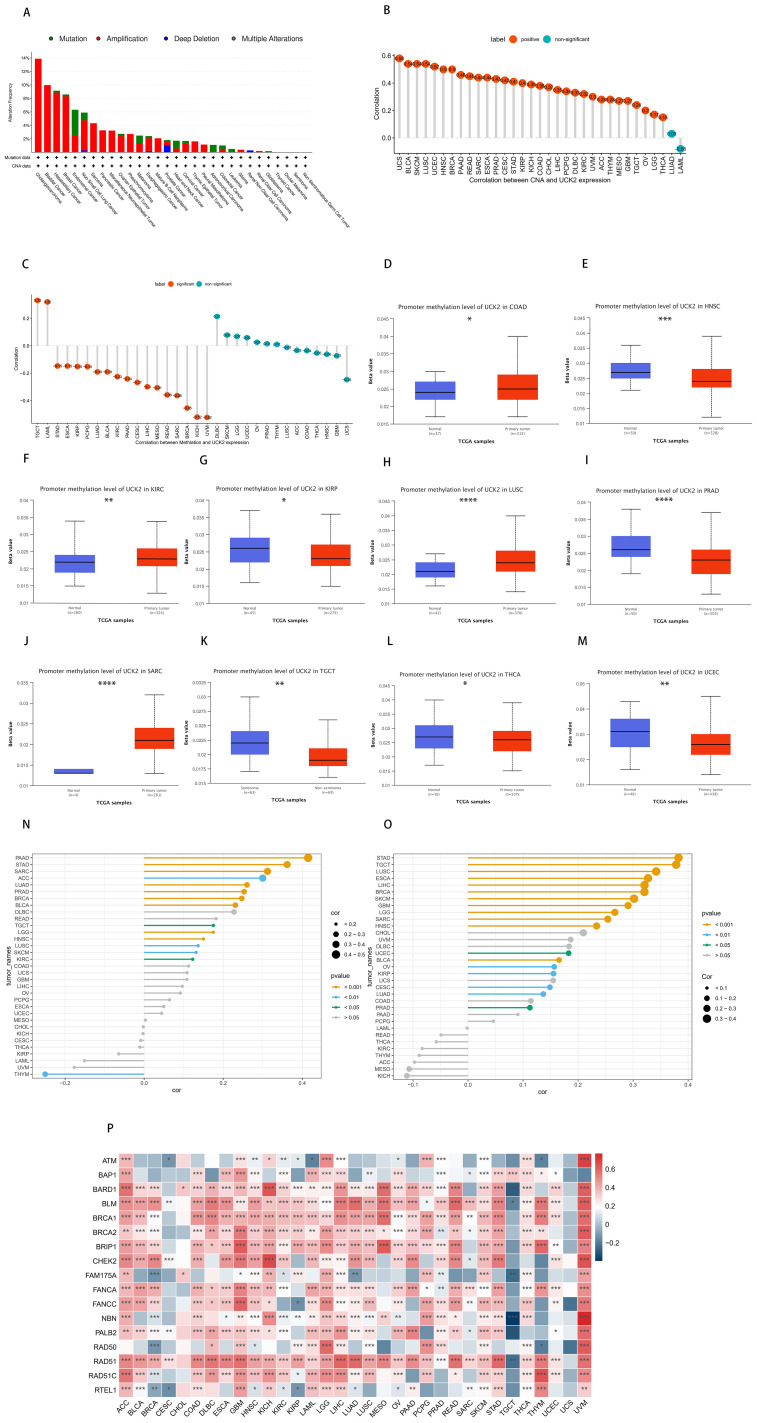
In tumors, the gene UCK2 is associated with genetic instability. **(A)** The genomic alterations of UCK2, including Mutation (Missense mutation, Truncating mutation, Inframe mutation, Splice mutation, Fusion mutation), Amplification, Deep Deletion, and Multiple Alterations (copy number alteration), were analyzed in ICGC/TCGA pan-cancer studies. **(B)** Pearson’s correlation analysis between UCK2 expression and CNA, using data from the TCGA dataset. **(C)** Pearson’s correlation analysis of UCK2 expression and DNA methylation, using TCGA dataset. **(D-M)** A comparison of the promoter DNA methylation status of UCK2 was conducted between cancer tissues and adjacent normal tissues across multiple types of cancer, including **(D)** COAD, **(E)** HNSC, **(F)** KIRC, **(G)** KIRP, **(H)** LUSC, **(I)** PRAD, **(J)** SARC, **(K)** TGCT, **(L)** THCA, **(M)** UCEC. **(N)** The relationship between UCK2 expression and **(N)** TMB and **(O)** MSI was examined. **(P)** The heatmaps illustrate the correlation between UCK2 expression and HRD. *p<0.05, **p<0.01, ***p<0.001, ****p<0.0001.

Gene mutations can significantly impact the modulation of tumor growth and progression. We used cBioPortal (https://www.cbioportal.org/) to analyze the relationship between UCK2 expression and mutations, as well as copy-number alterations (CNAs). The mutation status of UCK2 in various cancers was evaluated and is depicted in (**Figure 4A**), we found that high UCK2 amplification in most cancers, especially cholangiocarcinoma, bladder cancer. According to **Figure 4A**, deep deletion is present in non-small cell lung cancer, esophageal-gastric cancer, prostate cancer (highest), and renal non-clear cell carcinoma, as indicated by the blue color. Moreover, UCK2 expression showed a positive correlation with CNV in 31 out of 33 cancers, with the exception LUAD and LAML (**Figure 4B**). We found that the gene UCK2 was highly expressed in LUAD, but it was not associated with copy number variation in LUAD. This phenomenon can be attributed to various complex biological mechanisms. One possible explanation is that even within the same cancer type, genetic backgrounds, environmental factors, and tumor heterogeneity can all influence the relationship between gene expression and copy number variation. For example, if a gene has other regulatory factors involved in its expression control, such as transcription factors or microRNAs, changes in copy number may not necessarily affect its expression levels. Furthermore, some cancers may alter gene expression through non-genetic mechanisms such as DNA methylation, histone modifications, and other epigenetic processes, which are independent of copy number variation. This could explain why there is no correlation between UCK2 expression and copy number variation in LUAD, despite the significant difference compared to normal tissue.

DNA methylation is a crucial chemical modification that plays a pivotal role in the regulation of epigenetic gene expression (67–69). In this study, our observations indicated that in 18 out of 32 types of cancers, there were statistically significant findings. Among these 18 cancers, the expression of UCK2 exhibited a negative correlation with DNA methylation levels in 16 cancers, while in 2 cancers, it showed a positive correlation. However, this correlation was found to be absent in several cancers, including DLBC, SKCM, LGG, UCEC, OV, PRAD, THYM, LUSC, ACC, COAD, THCA, HNSC, GBM, and UCS (as illustrated in **Figure 4C**). Studies have shown that in LUSC, DNA hypomethylation promotes the high expression of pyrimidine metabolism rate-limiting enzymes (70), our study also found this negative correlation, but it was not statistically significant, which was contrary to our study. In another study on liver cancer prognosis, DNA methylation was negatively correlated with UCK2 expression, which obtained the same result as ours (71) Additionally, our study has revealed that the DNA methylation level of the UCK2 promoter is significantly reduced in UCEC, THCA, TGCT, PRAD, KIRP, and HNSC, while it is elevated in SARC, LUSC, KIRC, and COAD, in comparison to the adjacent normal tissues (**Figures 4D–M**). Thus, the observed abnormal increase in UCK2 mRNA expression might be linked to genetic alterations and decreased levels of DNA methylation. Moreover, in this study, we have conducted a comprehensive analysis of the correlations between TMB, MSI and HRD with UCK2 in pan-cancer. These genomic alterations are commonly observed in various types of cancers and have been shown to significantly impact patient prognosis and therapeutic responses (72, 73). As showed in **Figure 4N**, UCK2 demonstrated a positive correlation with TMB in 14 tumor types, namely PAAD, STAD, SARC, ACC, LUAD, PRAD, BRCA, BLCA, DLBC, READ, TGCT, LGG, HNSC, LUSC, SKCM, and KIRC and with MSI in 18 tumors (STAD, TGCT, LUSC, ESCA, LIHC, BRCA, SKCM, GBM, LGG, SARC, HNSC, UCEC, BLCA, OV, KIRP, CESC, LUAD and PRAD) (**Figure 4O**). We discovered that UCK2 was positively correlated with multiple HRD genes in most cancers, including ACC, GBM, KICH, LGG, LIHC, LUSC, MESO, PAAD, PCPG, READ, SKCM, STAD THCA, and especially UVM (**Figure 4P**). It has been reported that HRD is ubiquitous in a variety of cancers, and ATM and BRCA1/2 have been found to be important mutation drivers (74).

### UCK2 correlates with DNA repair and stemness in cancer

The maintenance of genomic stability in cancer primarily relies on the repair of DNA through various mechanisms, such as DNA MMR (52, 75) and HRR, which also play a role in preserving stemness in cancers (26, 76, 77). Therefore, we conducted an analysis of the associations between UCK2 expression and MMR-related genes, HRR signature, and tumor stemness scores (DNA stemness scores and RNA stemness scores.

**Figure 5:**
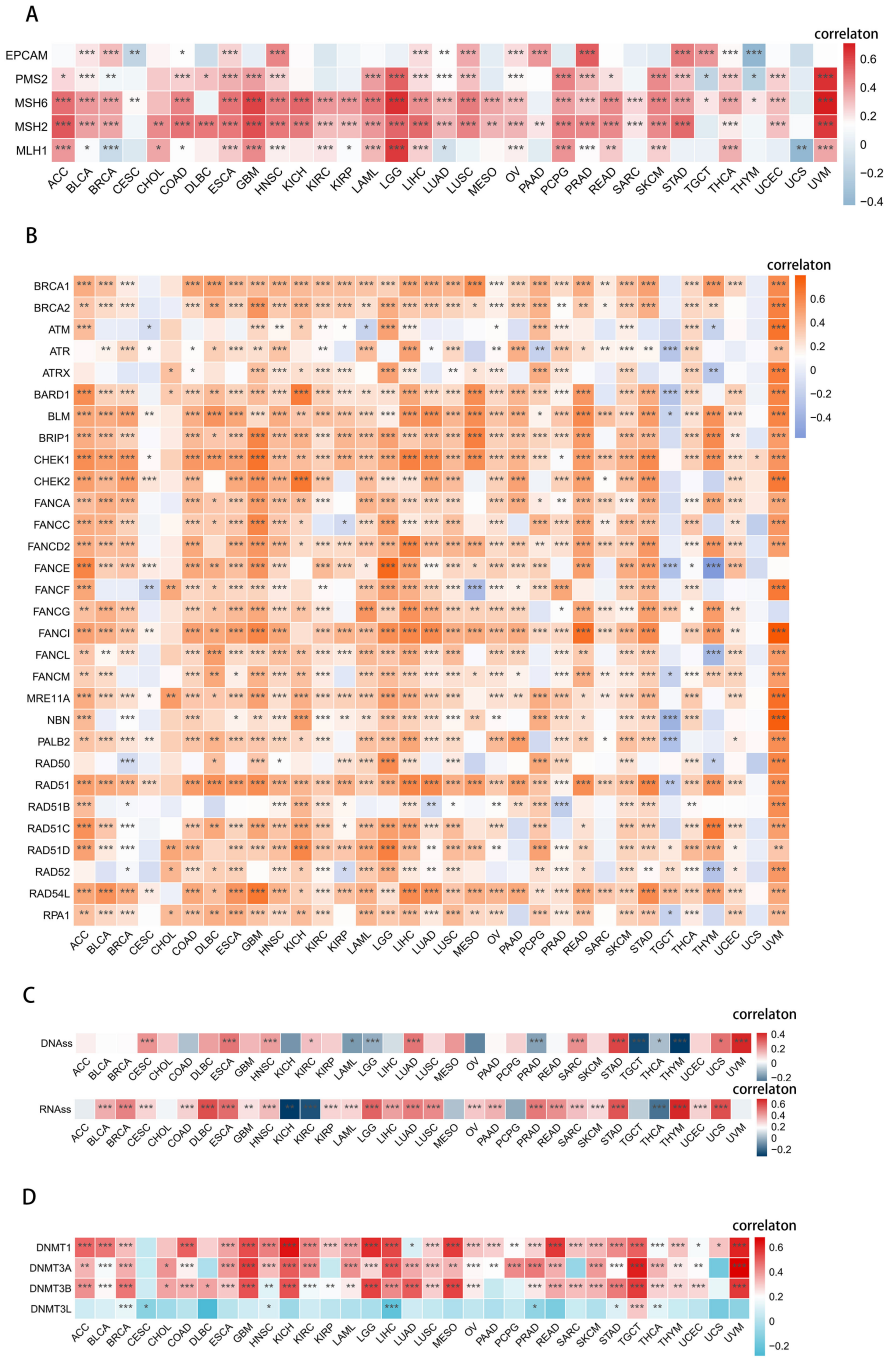
UCK2 was found to be involved in DNA repair, stemness, and epigenetic modulations in various types of cancers. **(A)** Heatmaps to show the relationship between UCK2 and 5 MMR genes in pan-cancer [these 5 genes (MLH1, MSH2, MSH6, PMS2, EPCAM) are the main genes in the MMR system (78)]. **(B)** Heatmaps to show the relationship between UCK2 expression and 30 genes’ HRR in pan-cancer. **(C)** Spearman correlation analysis was conducted for DNAss [DNAss. DNA methylation-based (Stem cell signature probes (219 probes), that combines the 3 signatures listed below. This score will drive the main figures in the PancanAtlas paper. EREG-METHss. Epigenetically regulated DNA methylation-based (87 probes). DMPss, Differentially methylated probes-based (62 probes); ENHss, Enhancer Elements/DNAmethylation-based (82 probes)], RNAss [RNAss, RNA expression-based (All set of available genes) this score will drive the main figures in PancanAtlas paper. EREG.EXPss, Epigenetically regulated RNA expression-based (103 genes)], and UCK2 gene expression. **(D)** The heatmaps exhibits the relationship between UCK2 and 4 methyltransferases in pan-cancer. *p < 0.05, **p < 0.01, ***p < 0.001.

Our analysis has revealed significant positive correlations between UCK2 expression and MMR-related genes in most cancers, particularly in UVM, as evidenced by (**Figure 5A**). However, we did not observe this correlation in CESC, CHOL and UCS, Interestingly, we also found positive correlations between UCK2 expression and HRR signature in most cancers, including UVM, as shown in (**Figure 5B**). It appears that UCK2 expression plays an important role in DNA damage repair.

We found a significant positive association between RNA stemness scores (RNAss) and UCK2 expression in most cancers, as shown in (**Figure 5C**). Similarly, we also found that DNA stemness scores (DNAss) were positively correlated with UCK2 expression in 9 tumors, including CESC, ESCA, HNSC, KIRC, LUAD, SARC, STAD, UCS, and especially UVM, (as depicted in **Figure 5C**). Overall, this illustrates that the higher expression of UCK2 is associated with a higher tumor stemness score, stronger the activity of tumor stem cells, and lower the degree of tumor differentiation.

Various cancers show coordinated expression of DNA methyltransferases (DNMTs), as reported (79). We investigated the negative correlation between UCK2 expression and DNMTs in CESC, LIHC and PRAD (**Figure 5D**). While a positive association between UCK2 expression and DNMTs in 30 other cancers. Notably, UVM, TGCT, KICH, and GBM showed the most significant positive correlation. These results suggest that high UCK2 expression may promote DNMTs expression in diverse types of cancer.

### Enrichment analysis of UCK2-related genes

**Figure 6:**
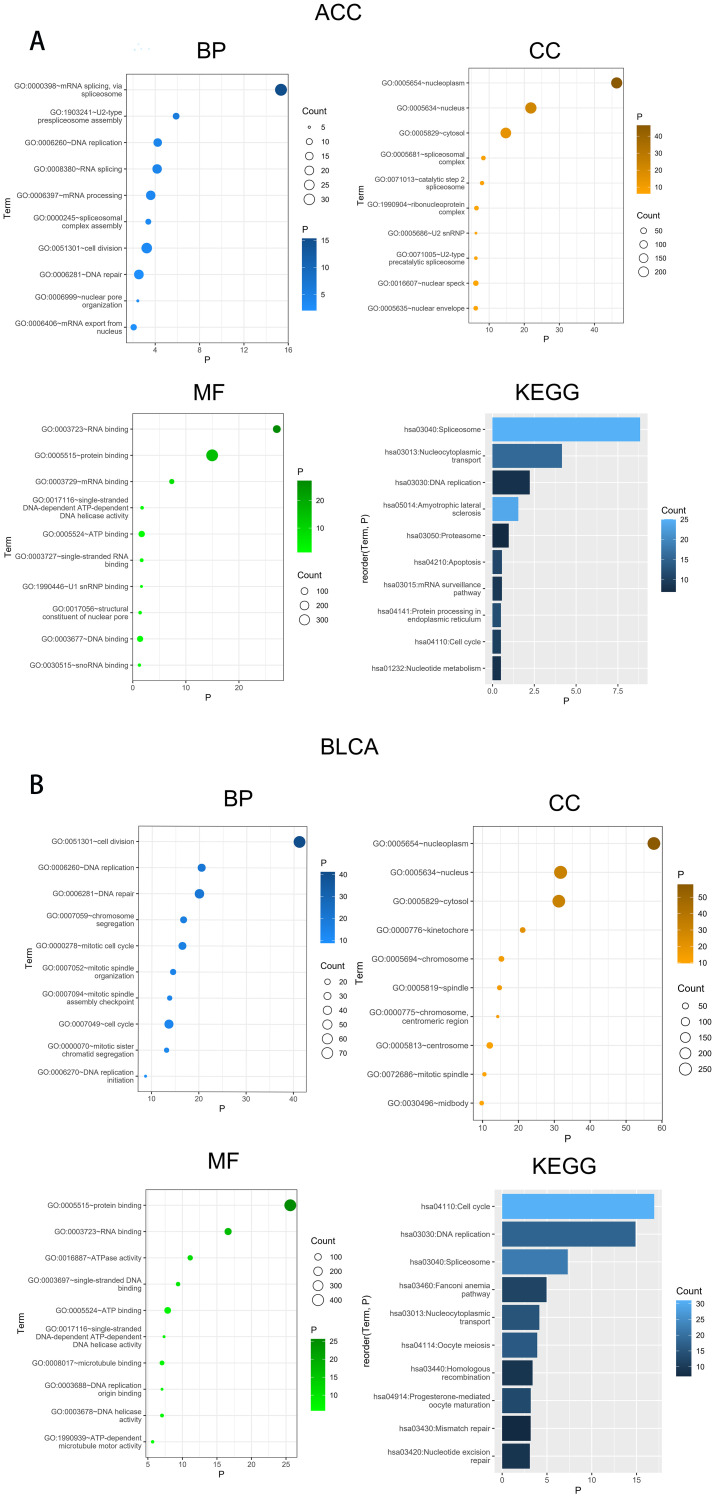
Results of Gene Ontology (GO) term and Kyoto Encyclopedia of Genes and Genomes (KEGG) pathway enrichment analysis in **(A)** ACC, **(B)** BLCA.

To determine the potential function and pathway of UCK2, we conducted the GO and KEGG analysis. In BLCA, UCK2 showed significant correlated with biological processes such as cell division, DNA replication and DNA repair. Additionally, UCK2 was found to be primarily located in the nucleoplasm, nucleus and cytosol. Its most associated molecular functions were protein binding, RNA binding and ATPase activity. The signing pathway for UCK2 was identified as involving cell cycle, DNA replication and spliceosome. Cancers with similar biological functions to UCK2 in BLCA include COAD, ESCA, DLBC, HNSC, LAML, LGG, LIHC, LUAD, LUSC, MESO, READ, STAD, ACC, and THYM (**Figures 6A, B**; **Supplementary Figures S7**–**S13**).

**Figure 7:**
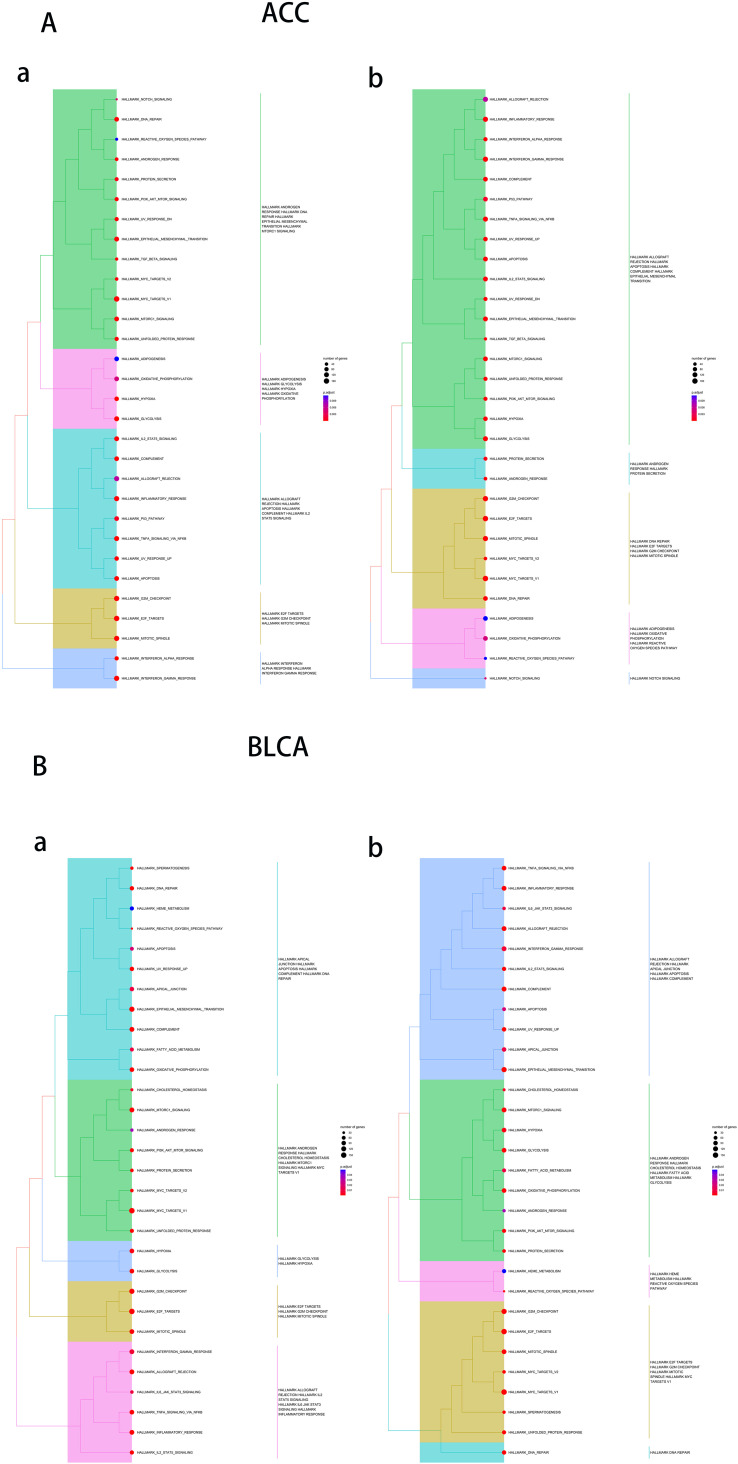
Results of gene set enrichment analysis (GSEA) in **(A)** ACC, **(B)** BLCA. In figure a, the default values for hclust_method are used, in figure b, “average” is used as the hclust_method.

To further investigate the biological processes associated with UCK2, we conducted GSEA using hallmark gene sets. The main enriched biological functions of UCK2-related genes include DNA repair, glycolysis, E2F target, and G2M checkpoint, which are observed in several types of tumors, including ACC, BLCA, BRCA, COAD, DLBC, ESCA, HNSC, LAML, LGG, LIHC, LUAD, LUSC, MESO, READ, STAD and GBM. We also have observed that UCK2 is associated with interferon-gamma (IFNγ) response in several tumors such as ACC, BRCA, DLBC, and COAD. Previous studies have reported that IFNγ produced by T cells converts non-cancer stem cell to cancer stem cells (80). These findings suggest that UCK2 may play an essential role in maintaining genomic stability, regulating cellular metabolism, and controlling cell cycle progression in tumorigenesis, and adjusting the immune response to maintain stemness in tumor as shown in (**Figures 7A, B**; **Supplementary Figures S14**–**S21**).

### Exploring the correlation between UCK2 expression and immune cell infiltration

**Figure 8:**
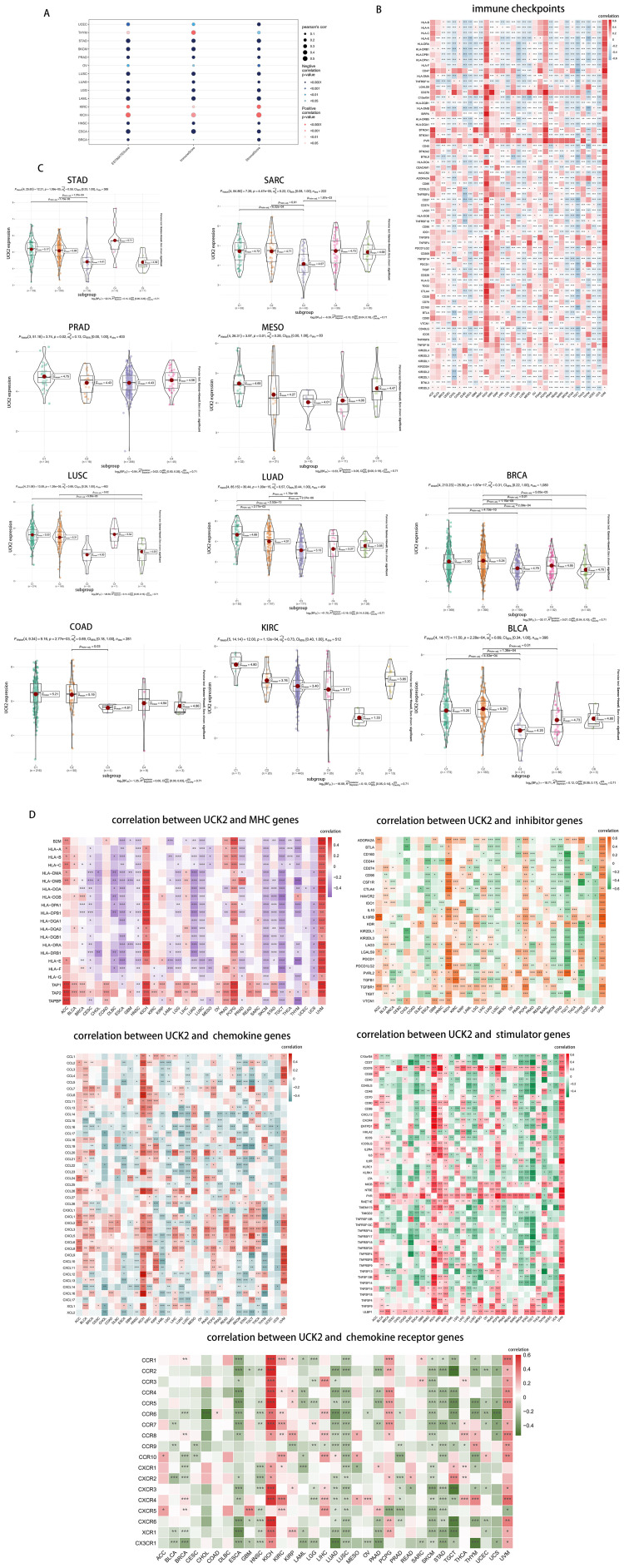
UCK2 was found to be inversely correlated with immune infiltration in pan-cancer studies. **(A)** The correlations between UCK2 and ESTIMATEScore, ImmuneScore, and StromalScore are presented. **(B)** The heatmaps to show the relationship between UCK2 expression and immune-checkpoint-associated genes. **(C)** The expression of UCK2 across 6 immune subtypes in 10 different types of cancer **(D)** The heatmaps of the correlations between UCK2 expression and MHC genes, inhibitors genes, chemokine genes, stimulator genes, and chemokine receptor genes are displayed. *p < 0.05, **p < 0.01, ***p < 0.001.

According to reports, UCK2 genes has been found to enhance the immune response in cases of hepatocellular carcinoma (43). Thus, we calculated the ESTIMATE of UCK2 in various types of cancer. **Figure 8A** illustrates that UCK2 consistently showed a negative correlation with the ESTIMATEScore, ImmuneScore, and StromScore in tumors, such as UCEC, STAD, SKCM, PRAD, OV, LUSC, LUAD, LGG, LAML, HNSC, ESCA, and BRCA. However, there was a positive correlation in KICH and KIRC. In THYM alone, UCK2 expression presented a positive correlation with the ESTIMATEScore, ImmuneScore, but a negative correlation with the StromScore. Some tumor cells can exploit immune checkpoint molecules to evade immune attacks. Thereby, increasing their chances of survival and metastasis (81). The cancers showing significant negative correlation between UCK2 expression and various immune-checkpoint-associated genes, including CHOL, DLBC, ECCA, LUSC, SKCM, TGCT, THYM, and UCS. Especially, in multiple types of cancers, a strong correlation was observed between UCK2 expression and human leucocyte antigen (HLA), mainly including HLA-I and HLA-II (**Figure 8B**). Several studies have documented the involvement of HLA molecules in promoting immune evasion by tumors (82–84). These findings suggest that the UCK2 gene may play a role in the interplay between immune checkpoints and tumor development. We Subsequently investigated whether there were differential expressions of UCK2 across various cancer immune subtypes. Our results revealed notable associations between UCK2 expression and immune subtypes in ten cancers (**Figure 8C**). It was observed that UCK2 expression was comparatively higher in the C4 immune subtype (Lymphocyte Deplete) when compared to other subtypes in several cancers, such as LUAD, STAD, SARC, and LUSC. Interestingly, there was an increase in UCK2 expression in the C2 immune subtype (IFN-gama Dominant) for BLCA and BRCA. These findings suggest that UCK2 may potentially promote tumor progression by affecting lymphocyte functions.

Furthermore, our analysis included the examination of correlations between UCK2 expression and key genes related to major histocompatibility complex (MHC), immune activation, immune suppression, chemokines, and chemokine receptors across various cancer types. As depicted in heatmaps (**Figure 8D**), UCK2 exhibited a positive correlation with genes related to five gene families in different tumor types, namely ACC, KICH, PCPG, KIRC, and UVM. This correlation was particularly significant across all five gene families, with an overall positive correlation. In contrast, it showed an overall negatively correlated with genes related to MHC in several tumors, including DLBC, ESCA, HNSC, LUSC, SKCM, TGCT, and UCS. It also exhibited negative correlations with genes related to immunoinhibitors in ESCA, LUSC, SKCM, STAD, and TGCT, with chemokine receptors in ESCA, HNSC, LUAD, LUSC, SKCM, STAD, and TGCT, with immunostimulatory genes in ESCA, LUAD, LUSC, SKCM, STAD, and TGCT, and with chemokines in ESCA, LUAD, LAML, LUSC, SKCM, and PRAD. Overall, these results suggest a close association between UCK2 expression and the biological function of immune-related genes.

### Unveiling UCK2 expression in tumor and immune cells in pan-cancer

**Figure 9:**
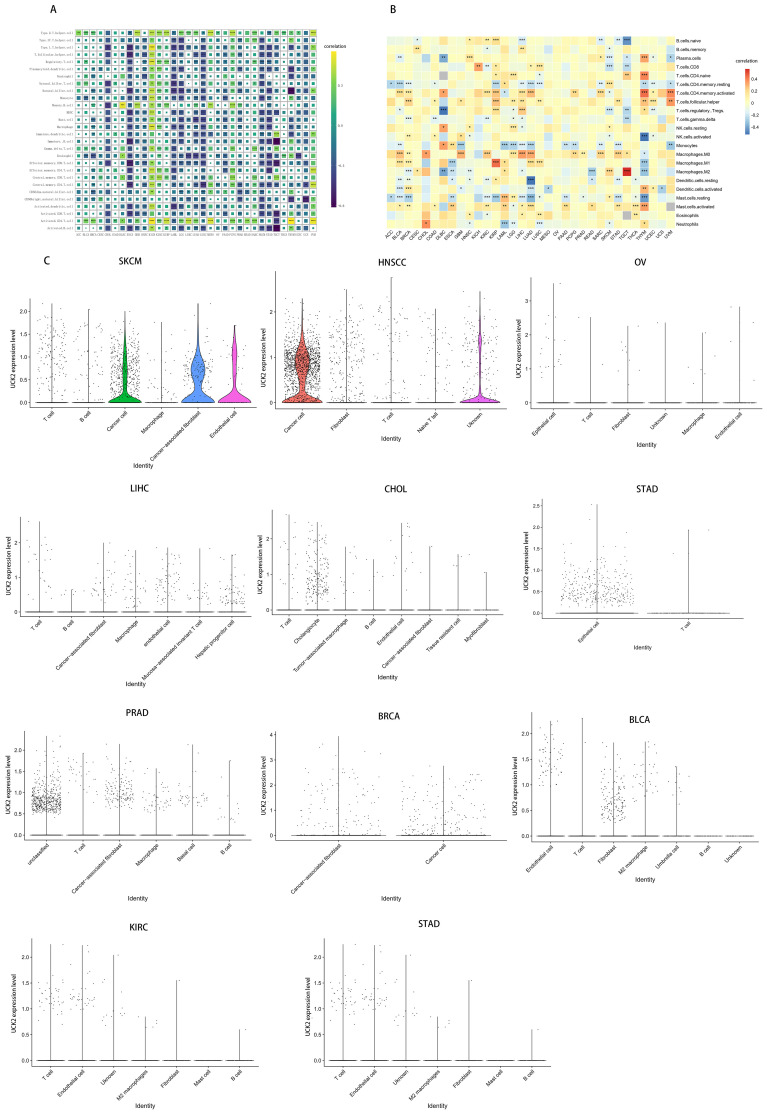
UCK2 was found to be associated with immune cell infiltration in a variety of tumor types. **(A)** Correlation between UCK2 and immune gene markers genes **(B)** CIBERSORT was used to calculate immune cell infiltration in pan-cancer. **(C)** The violin plots illustrating the expression of UCK2 in single-cell cluster across various types of cancer. *p < 0.05, **p < 0.01, ***p < 0.001.

To further explored the UCK2 expression in cancer immunity, we conducted a study investigating its correlation with the level of infiltration of 28 immune cell types by the ssGSEA method and Hmisc function (**Figure 9A**). our findings revealed strong positive correlations between UCK2 expression and T helper type 2 cells, activated CD4+ T cells, central memory CD8+ T cells, and memory B cells across multiple types of cancer. Conversely, we observed a significant negative correlation between UCK2 expression and T helper type 1 cells, T follicular helper cells, nature killer cells, monocytes, myeloid-derived suppressor cells (MDSCs), immature dendritic cells, immature B cells, eosinophils, effector memory CD8+ T cells, central memory CD4+ T cells, CD56bright natural killer cells, activated dendritic cells, activated CD8+ T cells, macrophages, and activated B cells within different cancer types.

Additionally, we employed the CIBERSORT algorithm to obtain 22 immunocyte correlations with UCK2. As illustrated in **Figure 9B**, our results indicated a positive correlation between UCK2 expression and activated memory CD4+ T cells, T follicular helper cells, M0 macrophages, M1 macrophages, and activated mast cells across several cancers. In contrast, we observed negative correlations between UCK2 expression and resting memory CD4+ T cells, monocytes, M2 macrophages, resting mast cells in various cancers. Unsurprisingly, these results were overall consistent with ssGSEA results with the aspect of T cells, monocytes, mast cells, and macrophages.

Furthermore, we conducted a comprehensive investigation into the expression of UCK2 on tumor and stromal cells in pan-cancer using the collected cancer single-cell datasets. Our findings revealed that UCK2 is significantly co-expressed on cancer cells and stromal cells in multiple cancer types, with particularly high expression observed on T cells, macrophages, cancer-associated fibroblasts, endothelial cells, cancer cells and fibroblasts (**Figure 9C**).

### Drug sensitivity analysis

**Figure 10:**
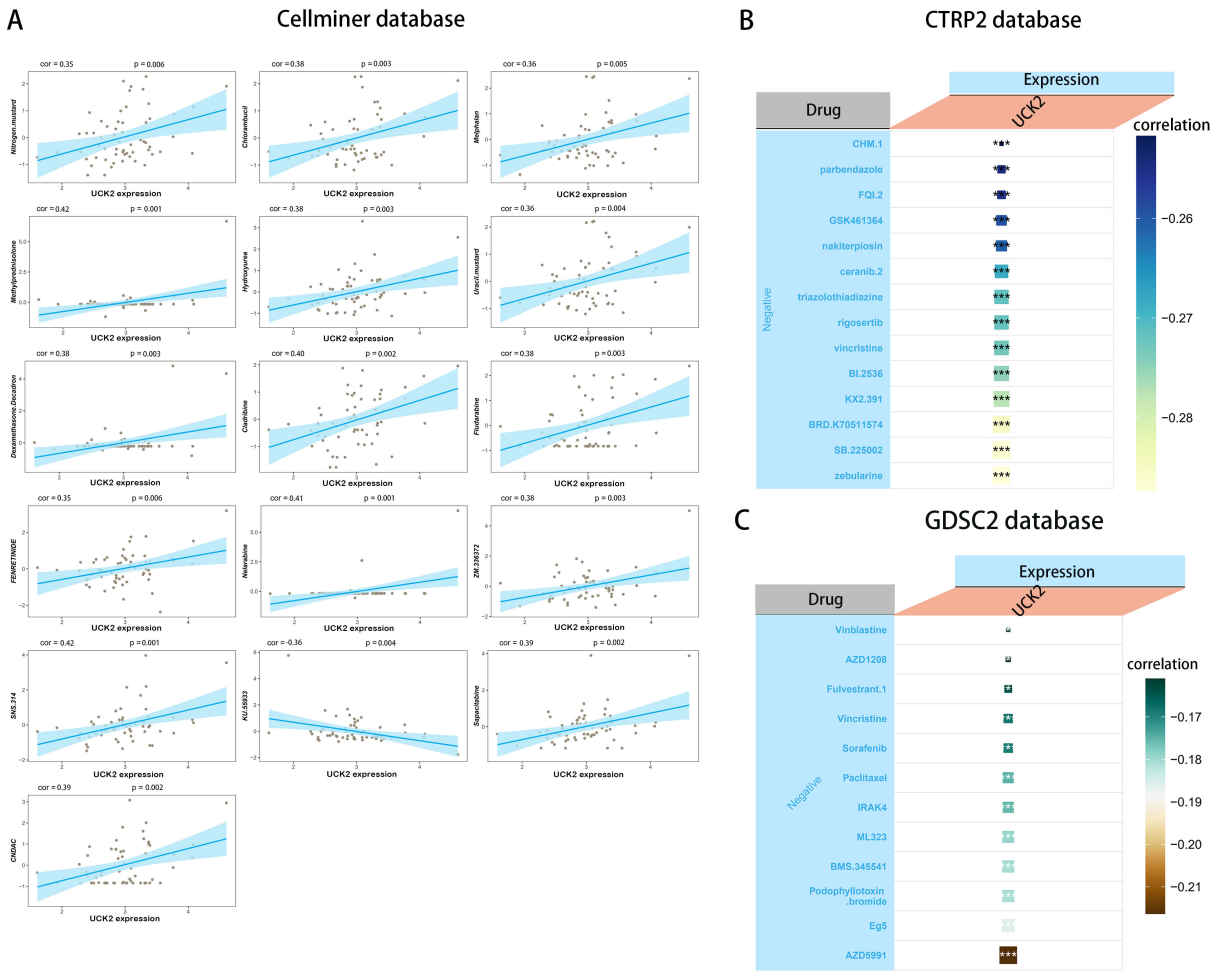
The relationship between UCK2 expression and drug sensitivity. **(A)** The expression of UCK2 was link to the sensitivity of Nitrogen mustard, Chlorambucil, Melphalan, Methylprednisolone, Hydroxyurea, Uracil mustard, Dexamethasone Decadron, cladribine, Fludarabine, FENRETINIDE, Nelarabine, ZM.336372, SNS.314, KU.55933, Sapacitabine, and CNDAC from the CellMiner database. **(B)** The expression of UCK2 was associated with the sensitivity of 14 drugs from CTRP2 database. **(C)** The expression of UCK2 was associated with the sensitivity of 12 drugs from GDSC2 database. *p<0.05, ***p<0.001.

Our study, which utilized the CELLMINER drug response data, found that there was a positive correlation between UCK2 expression and sensitivity to drugs such as nitrogen mustard, chlorambucil, melphalan, methylprednisolone, hydroxyurea, uracil mustard, dexamethasone decadron, cladribine, fludarabine, fenretinide, nelarabine, SNS-314, sapacitabine, and CNDAC. Conversely, UCK2 expression showed a negative association with susceptibility to KU-55933 (**Figure 10A**). Furthermore, we conducted a sensitivity analysis using the GDSC (85) and CTRP databases, which revealed that high UCK2 expression in patients led to susceptibility to the top five significant drugs: ML323, BMS-345541, podophyllotoxin bromide, Eg5, and AZD5991 in GDSC2 (**Figure 10C**) drug response data, and zebularine, SB-225002, BRD-K70511574, KX2-391, and BI-2536 in CTRP2 (**Figure 10B**) drug response data. These findings suggested that dysregulation of UCK2 could lead to anti-tumor drug resistance.

## Discussion

Recent research has placed significant emphasis on pan-cancer analysis of the whole genome, revealing crucial mutations, RNA alterations, and driver genes that play a pivotal role in cancer occurrence and progression, thereby holding immense importance for early diagnosis and effective biomarker development (78, 86–90). The heterogeneity of tumor presents significant challenges in their treatment (91). Pan-cancer analysis is a valuable method that can uncover intratumor heterogeneity (92, 93), thus offering valuable insights into cancer prevention strategies and the development of therapeutic targets. UCK2 is responsible for encoding a pyrimidine ribonucleoside kinase that plays a pivotal role in phosphorylating uridine and cytidine, converting them into uridine monophosphate (UMP) and cytidine monophosphate (CMP) respectively (14). However, the role of UCK2 in pan-cancer among humans have yet to identified. Therefore, we conducted an exploration of UCK2 expression in multiple types of cancers, as well as its impact on the tumor immune microenvironment and immunotherapy. Studies have shown that in hepatocellular carcinoma, down-regulation of UCK2 induces cell cycle arrest and activates the age-related secretory phenotype associated with the TNF-α NFκB signaling pathway to alter the tumor microenvironment. In addition, UCK2 is a biomarker of the immunosuppressive microenvironment. Down-regulation of UCK2 induces tumor cells to produce secretory phenotype, improve the microenvironment, and reduce the tumor microenvironment UCK2 remodeling metabolism can reduce the resistance of tumor cells to T-cell-mediated killing (43) Tatsuro Watanabe et al. suggested that gene UCK2 promotes the vigorous cell proliferation of normal T cells and ATL cells through pyrimidine biosynthesis (43). UCK2 expression may promote the proliferation and activation of T cells, thereby enhancing the anti-tumor immune response. In addition, UCK2 may also alter the tumor microenvironment by activating the TNF-αNF κB signaling pathway associated with aging secretion phenotype in other cancers, promoting tumor growth and its immune response.

We observed that compared to normal tissues in the TCGA database, the expression of UCK2 was up-regulated in various tumors, including BLCA, BRCA, CESC, CHOL, COAD, ESCA, GBM, HNSC, KIRC, KIRP, LGG, LICH, LUAD, LUSC, OV, PAAD, PCPG, PRAD, SARC, SKCM, STAD, UCEC and UCS. However, it was down-regulated in ACC, DLBC, KICH and THCA. To further validate UCK2 expression from the bioinformatics analysis, we conducted immunohistochemistry studies. The results of these studies were consistent with the bioinformatics analysis for BRCA, COAD, PRAD, OV, BLCA, PAAD, READ, STAD, THCA and LUAD. However, in GBM, KIRC and LICH, UCK2 expression was low, contradicting the bioinformatics analysis. The primary reasons could be that the normal samples corresponding to GBM, KIRC and LICH are from the GTEx dataset, or that bioinformatics analysis may make predictions based on gene expression data while immunohistochemistry experiments detect based on protein expression. To reconcile these discrepancies, several methods can be used for verification and validation. Unexpectedly, we also found that UCK2 is highly expressed in CSCC, EC, and Lymphadenoma, but this result requires further verification. Elevated UCK2 expression was strongly correlated with adverse prognostic outcome across multiple cancer types. Intriguingly, UCK2 was identified as a protective factor in specific cancer subtypes, including CHOL, DLBC, OV, STAD. Besides, we analyzed the UCK2 expression in different pathologic stages. The results of our study revealed significant differences in gene expression during the development of various cancers, leading us to consider the potential of UCK2 gene as diagnostic and prognostic markers in pan-cancer. Analyzing the expression patterns of the gene could enhance our ability to detect and predict cancer progression. Further research is necessary to fully comprehend the functions and mechanisms of UCK2 gene in cancer development and progression.

Cancer stem cells (CSCs) are a subpopulation of tumor cells with stem-like properties, playing a crucial role in tumor initiation and progression, therapy resistance, and disease relapse. Cancer stem cells are believed to be a small subset of cells that drive cancer growth, repopulation following injury and metastasis (94). These cells, which are estimated to comprise approximately 0.05-3% of the heterogeneous cancer mass, have the ability to thrive under hypoxic conditions (95). Furthermore, cancer stem cells have been identified as the primary target for therapeutic interventions in cancer treatment (96).

The compositions of DNA repair, such as MMR and HRR, play a vital role in preserving the stemness of cancer stem cell (97, 98). In our study, we have discovered a significant positive correlation between UCK2 expression and various factors such as TMB, MSI, HRD, HRR, and MMR in multiple cancer types, including STAD, SARC, LUAD, PRAD, BRCA, BLCA, LGG, HNSC, LUSC, and SKCM. Meanwhile, GO, KEGG and GSEA analysis demonstrated that UCK2 expression was strictly linked with MMR in many cancers. These findings might suggest that these two repair systems maintain cancer stemness in the aforementioned cancers by the gene of UCK2. UCK2 uses the DNA repair pathway (99) to become a therapeutic target for cancer through several pathways, such as accurate and timely DNA repair during the occurrence of HRR and MMR, which is essential to maintain genomic stability. Loss of function of UCK2 may lead to an increase in errors in the repair process, which can exacerbate genomic instability in cancer cells. UCK2 may interact with other DNA repair-related proteins. By influencing the function of these proteins, UCK2 can play an important role in the DNA damage response.

We found that UCK2 expression was associated with glycolysis by GSEA analysis method in many cancers. Numerous studies have demonstrated the pivotal role of glycolysis in preserving the stemness of cancer cells (100–102). These results make us noticed that UCK2 gene may play important role in preserving cancer stemness through regulating glycolytic metabolism.

DNA methylation is crucial for the maintenance of CSCs in different types of cancer, such as leukemia, lung, and colon stem cells (103–105). In our study, we noticed that UCK2 expression is significantly negative correlation with DNA methylation in several cancers. On the contrary, the expression of UCK2 is strongly positive association with methyltransferase in various cancers. Moreover, we discovered 2 UCK2-related compounds that act as DNA methylation inhibitors. These results suggested that UCK2 may influence CSCs by DNA methylation or methyltransferase. To determine if UCK2 involved aforementioned biomedical processes, we need to conduct further research to clarified its specific mechanism.

Many evidences suggest that components within the TME can reprogram tumor initiation, growth, invasion, metastasis, and therapy response (31, 106). ESTIMATE algorithm assesses the scores of stromal and immune cells in tumor through gene expression (55). We observed an overall significant negative correlation between UCK2 expression and the ImmuneScore, ESTIMATEScore, StromalStore in various cancers. Thus, UCK2 may play an important role in immune infiltration.

As expected, our analysis of immune checkpoints reveals a strong association between UCK2 and HLA, which is in line with the findings from our correlation analysis between UCK2 and MHC genes. Numerous cancer cells within the body express both MHC I and MHC II, with MHC II play a key role in antigen presentation of CD4+ T-lymphocytes, the significance of CD4+ T- lymphocytes in anti-tumor immunity is increasingly recognized and valued (107). This indicates that the UCK2 gene may exerts anti-tumor immunity effects through CD4+ T cells. Recent studies have shown that loss of HLA may affect response to immune checkpoint inhibitor (ICI) PD-1 and CTLA-4 therapy. As UCK2 is closely related to HLA, UCK2 may affect immunotherapy by regulating HLA, which also provides new possibilities for future immunotherapy. It seems that bioinformatics analysis opens a new door for our research, but it also has bias. Recent studies have shown that the analysis of TCGA data may bring some bias (108, 109), so we should be more cautious when analyzing data.

Meanwhile, UCK2 expression and MHC/immune activation/immune suppression/chemokines/chemokine receptors genes show a consistent overall trend across all types of tumors. These findings greatly advance our understanding of the vital role of UCK2 in immune infiltration. Specifically, our GSEA analysis revealed a significant association between UCK2 expression and IFNγ in four different types of cancer. Furthermore, our findings indicate a significant increase in UCK2 expression within the C2 immune subtype (IFN-gamma Dominant) compared to other subtypes. Previous studies have shown that IFNγ can induce tumor stemness, prompting us to explore whether high UCK2 expression drives or results from cancer stemness mediated by IFNγ. Interestingly, we identified major histocompatibility complex (MHC)-II related to gene with an exceptionally high association with UCK2 in various cancers. Expression of MHC-II by tumor cells has been seen in various cancers, such as melanoma (110), breast cancer (111), prostate cancer (112), classic Hodgkin lymphoma (113), glioma (114). Increased expression of MHC-II can be caused by IFN-γ, and it can promote tumor immune evasion (107, 115, 116). Thus, we speculated that UCK2 plays a role in mediating the upregulation of MHC II expression by IFN-γ, thereby preserving cancer stem cells.

Moreover, we found that UCK2 expression was strongly correlated with the kinds of types of infiltrating immune cells and immune stromal (T cells, monocytes, mast cells, and macrophages, cancer-associated fibroblasts, endothelial cells, cancer cells and fibroblasts). This indicated that it broader immune effects. The overexpression of UCK2 was found to be positively correlated with the IC50 value of drugs that inhibit DNA repair and replication, these drugs, which mainly come from the CellMiner database, include nitrogen mustard, hydroxyurea, fludarabine, nelarabine, sapacitabine, and CNDAC. Conversely, UCK2 expression showed a negative association with susceptibility to KU-5593, which is known to inhibit DNA repair. Furthermore, our analysis revealed a negative correlation between UCK2 expression and microtubule-related inhibitors, such as Vinblastine, Vincristine, Podophyllotoxin-bromide, Eg5, parbendazole, nakiterpiosin, and triazolothiadiazine in the GDSC2 and CTRP2 databases. These findings suggest that these drugs have the potential to prevent cancer progression. Overall, these findings provide valuable insights for clinical drug selection and patient prognosis.

This study has limitations as it relies on computational analysis of genomic data. Future studies should include *in vivo* and vitro experiments to better understand the functional mechanisms of UCK2. Furthermore, our study has limitations in establishing the connection between UCK2 and immunotherapy. Further validation through clinical trials and cell experiments is needed to determine the role of UCK2 in immunotherapy. Although our findings suggest that a correlation between aberrant UCK2 expression, immune cell infiltration, and prognosis of human cancers, it remains unclear whether UCK2 may directly influences patient survival through an immune response. We will conduct future *in vitro*, *in vivo* and clinical studies of UCK2-targeted therapy. *In vivo* studies, we will use patient-derived xenograft models that have been well validated in previous studies, for example, Ru Li et al. have been very successful in establishing a mouse model of breast cancer metastasis comparing the effects of volatile anesthetics and intravenous anesthetics (117), and we will use a larger clinical cohort to further validate these findings.

## Conclusion

In conclusion, we perform multi-omics pan-cancer analyses of UCK2 and explored the of UCK2 in gene mutation, TMB, MSI, clinical prognostic value, immune cell infiltration, and drug sensitivity. UCK2 may participate in MMR or HDR, glycolysis, DNA methylation or methyltransferase, to promote cancer stem cell.

## Data Availability

The original contributions presented in the study are included in the article/**Supplementary Material**. Further inquiries can be directed to the corresponding author/s.

## Glossary

TCGA: The Cancer Genome Atlas
TMB: tumor mutation burden
MSI: microsatellite instability
TME: tumor immune microenvironment
ACC: Adrenocortical carcinoma
CESC: Cervical squamous cell carcinoma and Endocervical adenocarcinoma
DLBC: Lymphoid neoplasm difuse large B-cell lymphoma
GBM: Glioblastoma multiforme
HNSC: Head and neck squamous cell carcinoma
LAML: Acute myeloid leukemia
LGG: Brain lower grade glioma
OV: Ovarian serous cystadenocarcinoma
PCPG: Pheochromocytoma and Paraganglioma
PRAD: Prostate adenocarcinoma
SARC: Sarcoma
THYM: Thymoma
UCEC: Uterine corpus endometrial carcinoma
UCS: Uterine carcinosarcoma
UVM: Uveal melanoma
BLCA: bladder urothelial carcinoma
BRCA: breast invasive carcinoma
CHOL: cholangiocarcinoma
COAD: colon adenocarcinoma
ESCA: esophageal carcinoma
HNSC: head and neck squamous cell carcinoma
KICH: kidney chromophobe
KIRC: kidney renal clear cell carcinoma
KIRP: kidney renal papillary cell carcinoma
LIHC: liver hepatocellular carcinoma
LUAD: lung adenocarcinoma
LUSC: lung squamous cell carcinoma
MESO: mesothelioma
PAAD: pancreatic adenocarcinoma
READ: rectum adenocarcinoma
SKCM: skin cutaneous melanoma
STAD: stomach adenocarcinoma
TGCT: testicular germ cell tumors
THCA: thyroid carcinoma
UVM: uveal melanoma. RCC, Renal Cell Carcinoma
ALL: Acute Lymphoblastic Leukemia
CLL: Chronic Lymphoblastic Leukemia
GBC: Adenocarcinoma
AA: Astrocytoma
ATRT: Atypical Teratoid Rhabdoid Tumor
NHL: B-cell, Non-Hodgkins
CML: Chronic Myelogenous Leukemia
BL: B-cell, Non-Hodgkins Burkitts
MB: Medulloblastoma
MCL: B-cell, Non-Hodgkins, Mantle Cell
MRT: Malignant Rhabdoid Tumor
HL: B-cell, Hodgkins
PM: Mesothelioma
ALCL: T-cell, Non-Hodgkins, Anaplastic Large Cell
LCC: Large Cell Carcinoma
MM: Multiple Myeloma
NB: Neuroblastoma
SLCL: Small Cell Lung Cancer.
