# Supplementary material for: Uridine-cytidine kinase 2 is correlated with immune, DNA damage repair and promotion of cancer stemness in pan-cancer
Source: Front Oncol. 2025 Jan 27;15:1503300. doi: 10.3389/fonc.2025.1503300 (PMC11807824; doi:10.3389/fonc.2025.1503300)
Supplement: Supplementary file 1 [file DataSheet1.zip › Supplementary Figures 1/Supplementary material.docx]

**Supplementary material**

**Supplementary Figure 1** Pan-cancer analysis of UCK2 expression. (A) Pearson’s correlation analysis was employed to investigate the relationship between UCK2 expression and various types of cancer, using data from the TCGA database. (B) Pearson’s correlation analysis was employed to investigate the relationship between UCK2 expression and normal human tissues, using data from GTEx database. (C) Pearson’s correlation analysis was employed to investigate the relationship between UCK2 expression and tumor tissues, using data from CCLE database. (D) The differential expression of UCK2 in paired normal and tumor tissues was analyzed using data from the TCGA database.

**Supplementary Figure 2** The differential expression of UCK2 and pathological stages in (A) TGCT, (B) LUSC, (C) LUAD, (D) KIRP, (E) HNSC. * p < 0.05, ** p < 0.01, *** p < 0.001, **** p < 0.0001.

**Supplementary Figure 3** The expression of UCK2 correlates with overall survival time (OS). Kaplan-Meier analysis of the relationship between UCK2 expression and OS in (A) ACC, (B) BLCA, (C) KIRP, (D) LIHC, (E) BRCA, (F) CESC, (G) MESO, (H) OV, (I) CHOL, (G) COAD, (K) PAAD, (L) PRAD, (M) DLBC, (N) HNSC, (O) SARC, (P) STAD, (Q) KICH, (R) KIRC, (S) THCA, (T) UVM.

**Supplementary Figure 4** Immunohistochemistry of UCK2 in pan-cancer tissue chips and Statistical analysis of staining intensity. (A) BLCA, (B) EC, (C) PAAD, (D) READ. * p < 0.05, ** p < 0.01, *** p < 0.001, **** p < 0.0001.

**Supplementary Figure 5** Immunohistochemistry of UCK2 in pan-cancer tissue chips and Statistical analysis of staining intensity. (A) STAD, (B) LUAD, (C) CSCC. * p < 0.05, ** p < 0.01, *** p < 0.001, **** p < 0.0001.

**Supplementary Figure 6** Immunohistochemistry of UCK2 in pan-cancer tissue chips and Statistical analysis of staining intensity. (A) GBM, (B) KIRC, (C) LIHC, (D) Lymphadenoma, (E) THCA. * p < 0.05, ** p < 0.01, *** p < 0.001, **** p < 0.0001.

**Supplementary Figure 7-13** Results of Gene Ontology (GO) term and Kyoto Encyclopedia of Genes and Genomes (KEGG) pathway enrichment analysis in COAD, DLBC, ESCA, HNSC, LAML, LGG, LIHC, LUAD, LUSC, MESO, READ, STAD, THYM.

**Supplementary Figure 14-21** Results of gene set enrichment analysis (GSEA) in BRCA, COAD, ESCA, DLBC, GBM, HNSC, LAML, LGG, LIHC, LUAD, LUSC, MESO, READ, STAD, THYM. In figure a, the default values for hclust_method are used, in figure b, “average” is used as the hclust_method.
