# Supplementary figures and images for: Uridine-cytidine kinase 2 is correlated with immune, DNA damage repair and promotion of cancer stemness in pan-cancer
Source: Front Oncol. 2025 Jan 27;15:1503300. doi: 10.3389/fonc.2025.1503300 (PMC11807824; doi:10.3389/fonc.2025.1503300)

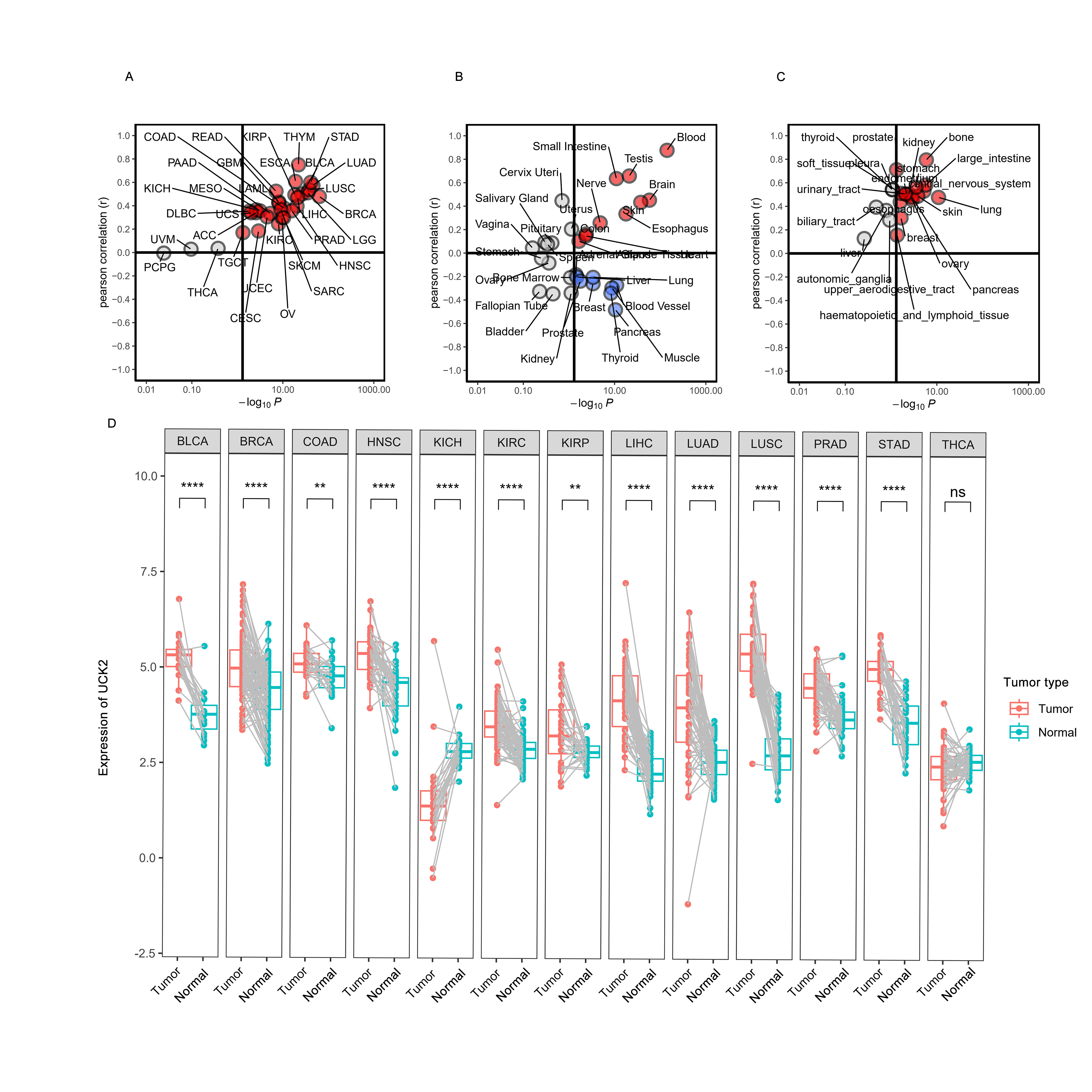

Supplement: Supplementary file 1 [file DataSheet1.zip › Supplementary Figures 1/Figure S1.jpg]

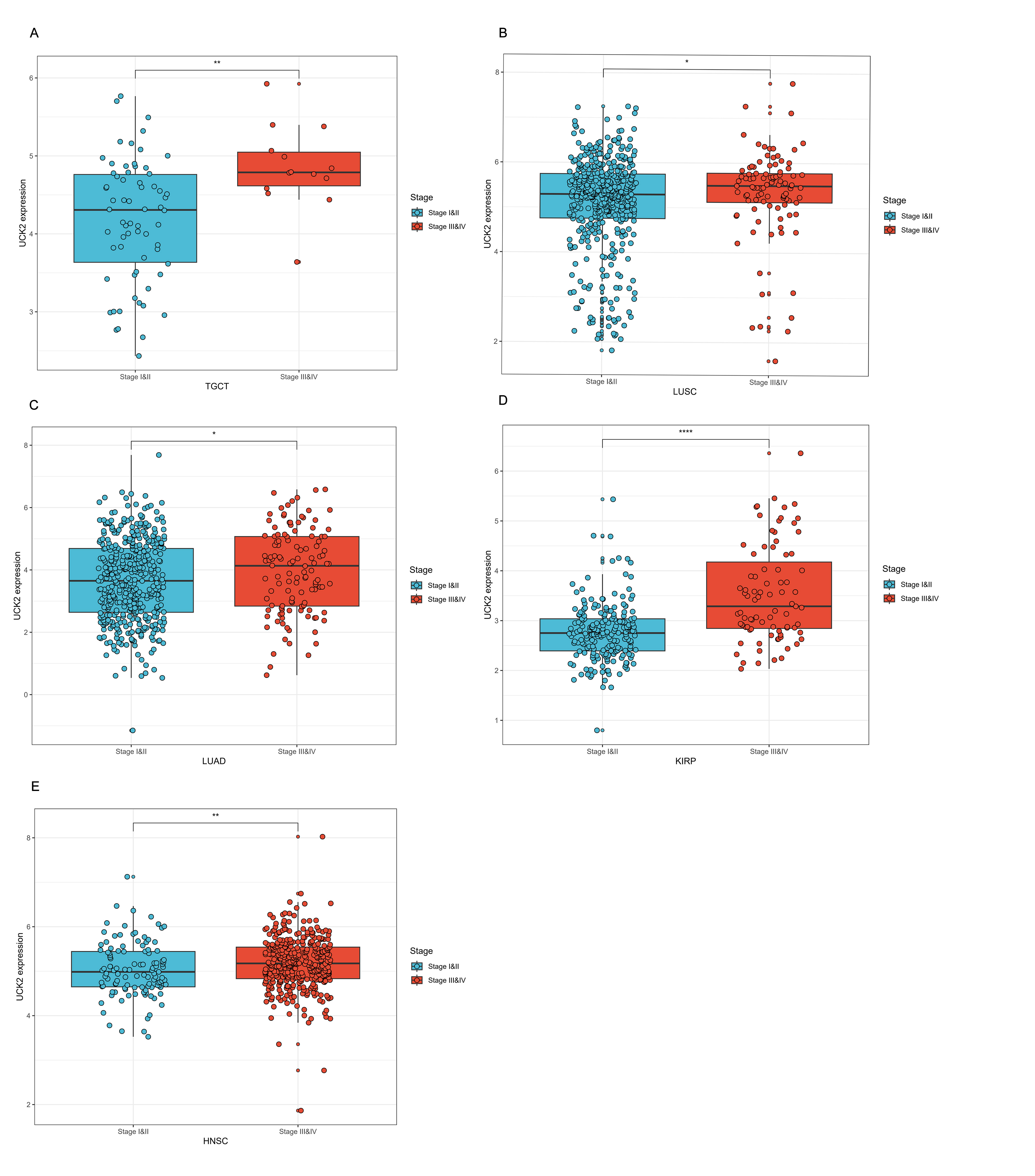

Supplement: Supplementary file 1 [file DataSheet1.zip › Supplementary Figures 1/Figure S2.jpg]

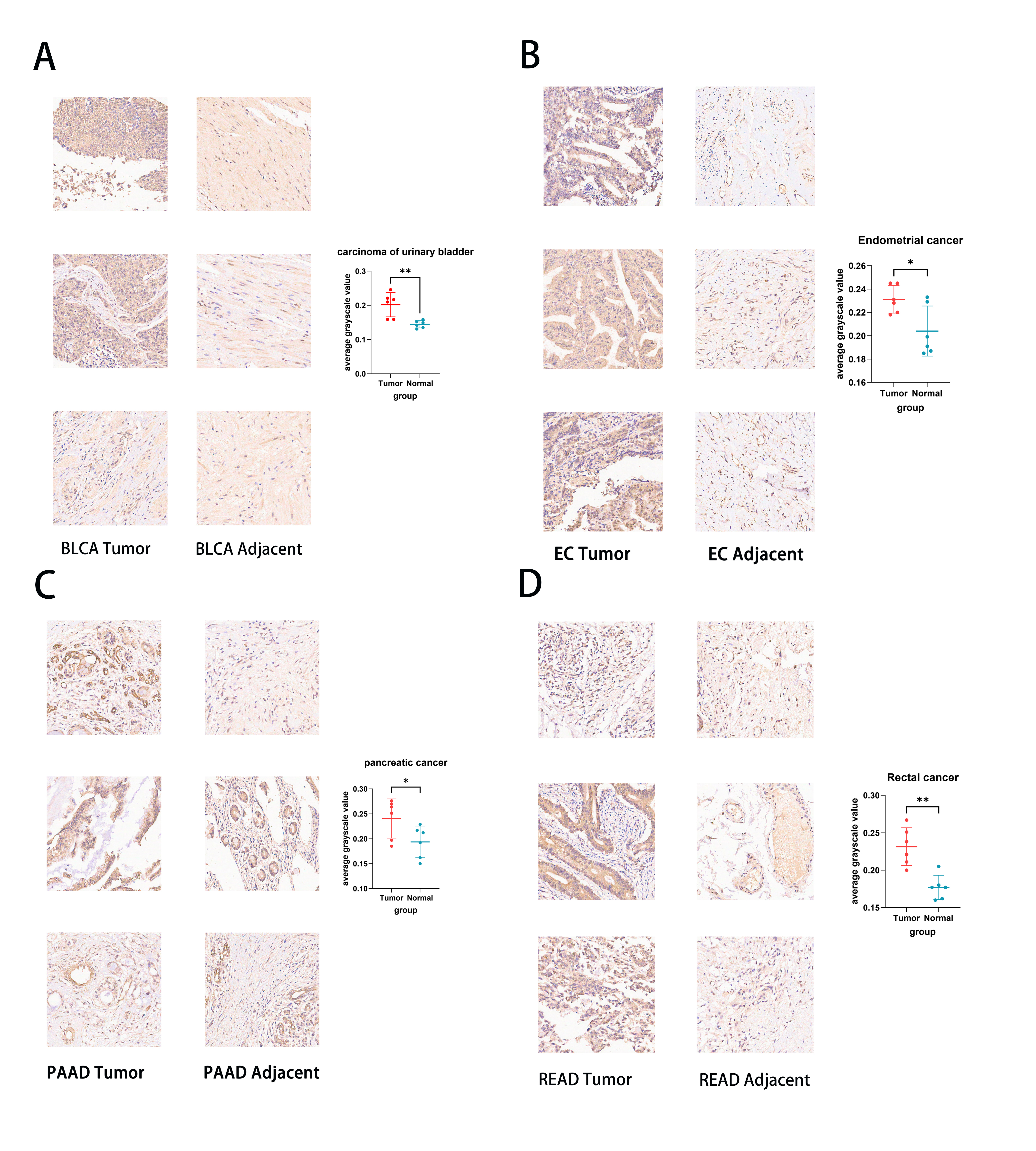

Supplement: Supplementary file 1 [file DataSheet1.zip › Supplementary Figures 1/Figure S3.jpg]

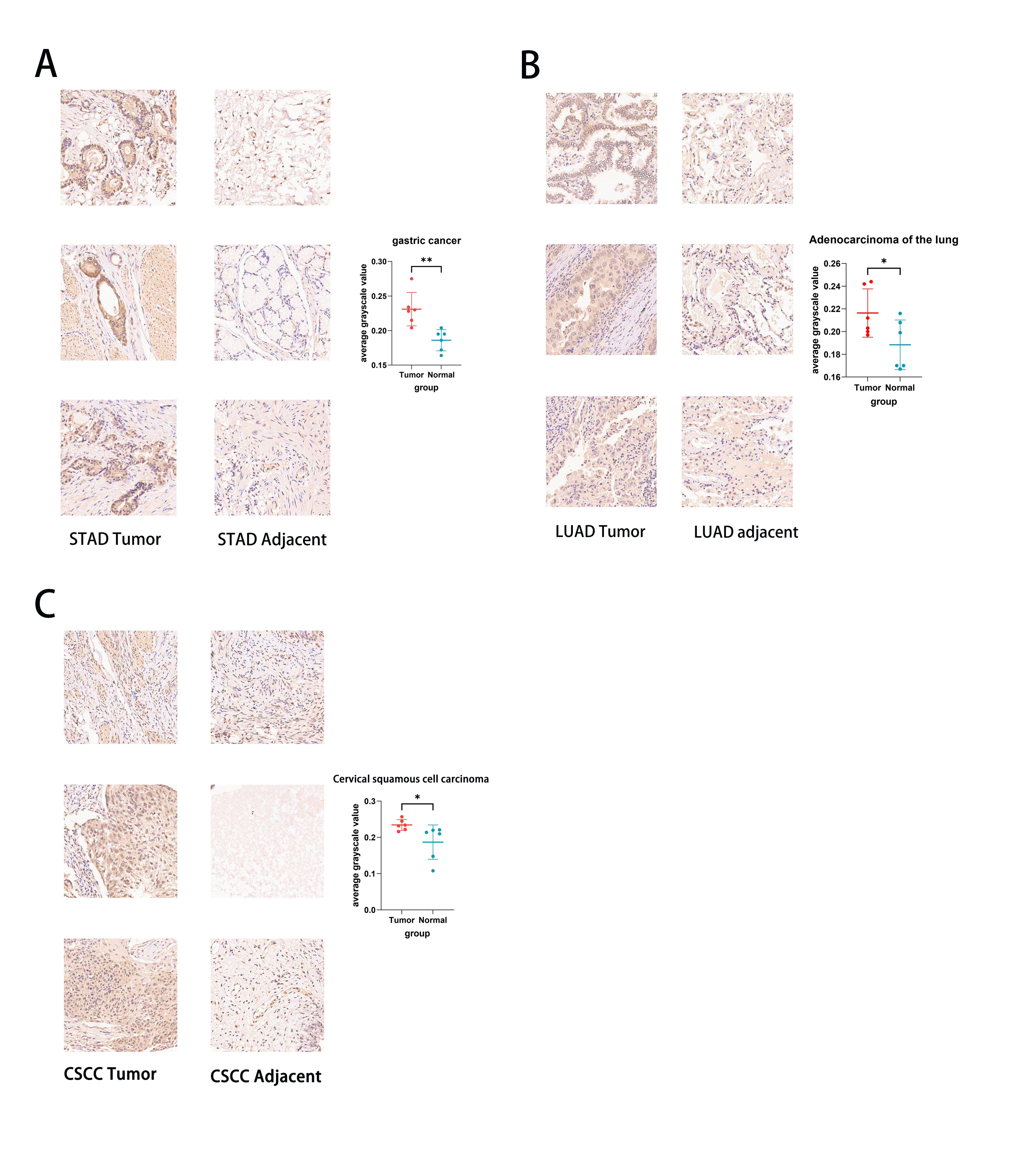

Supplement: Supplementary file 1 [file DataSheet1.zip › Supplementary Figures 1/Figure S4.jpg]

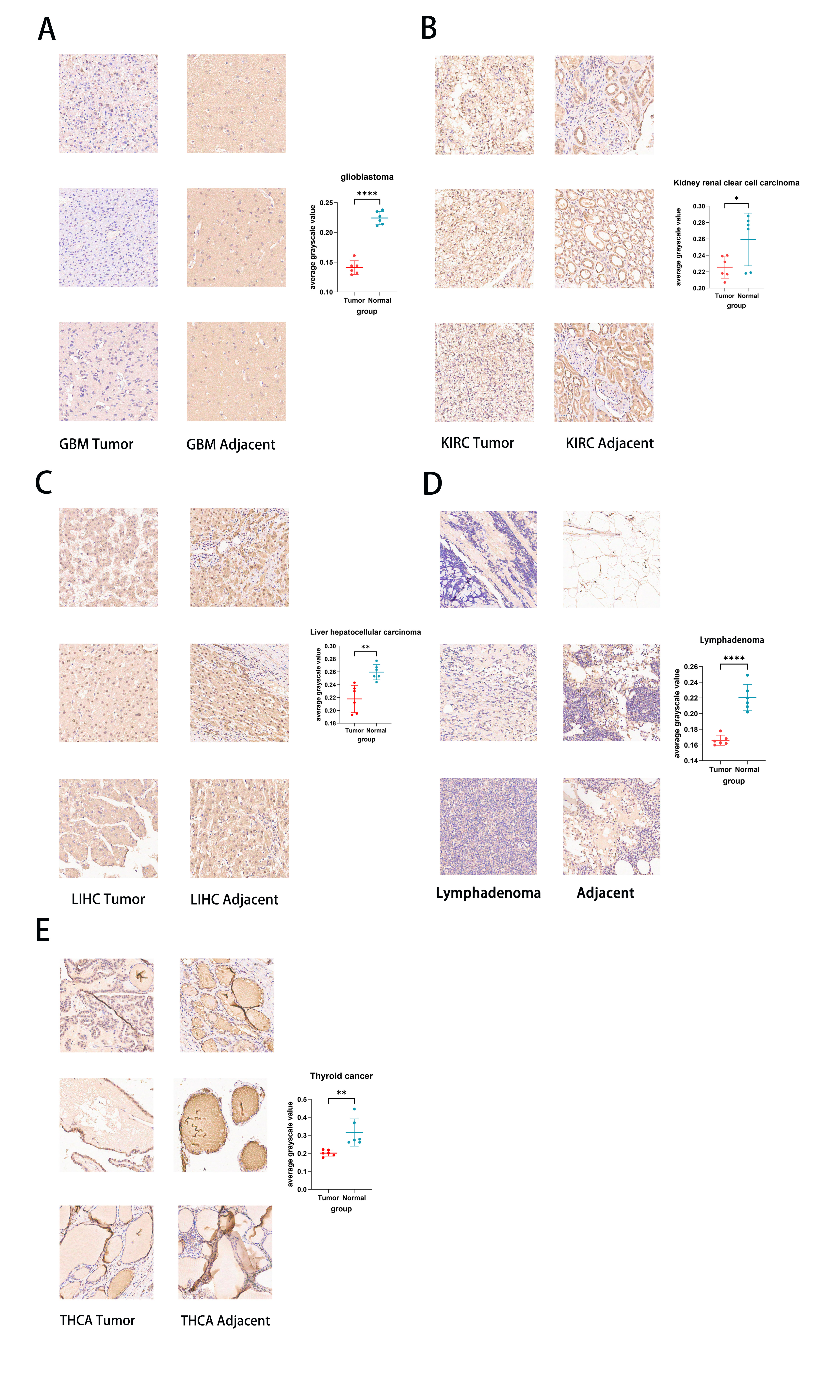

Supplement: Supplementary file 1 [file DataSheet1.zip › Supplementary Figures 1/Figure S5.jpg]

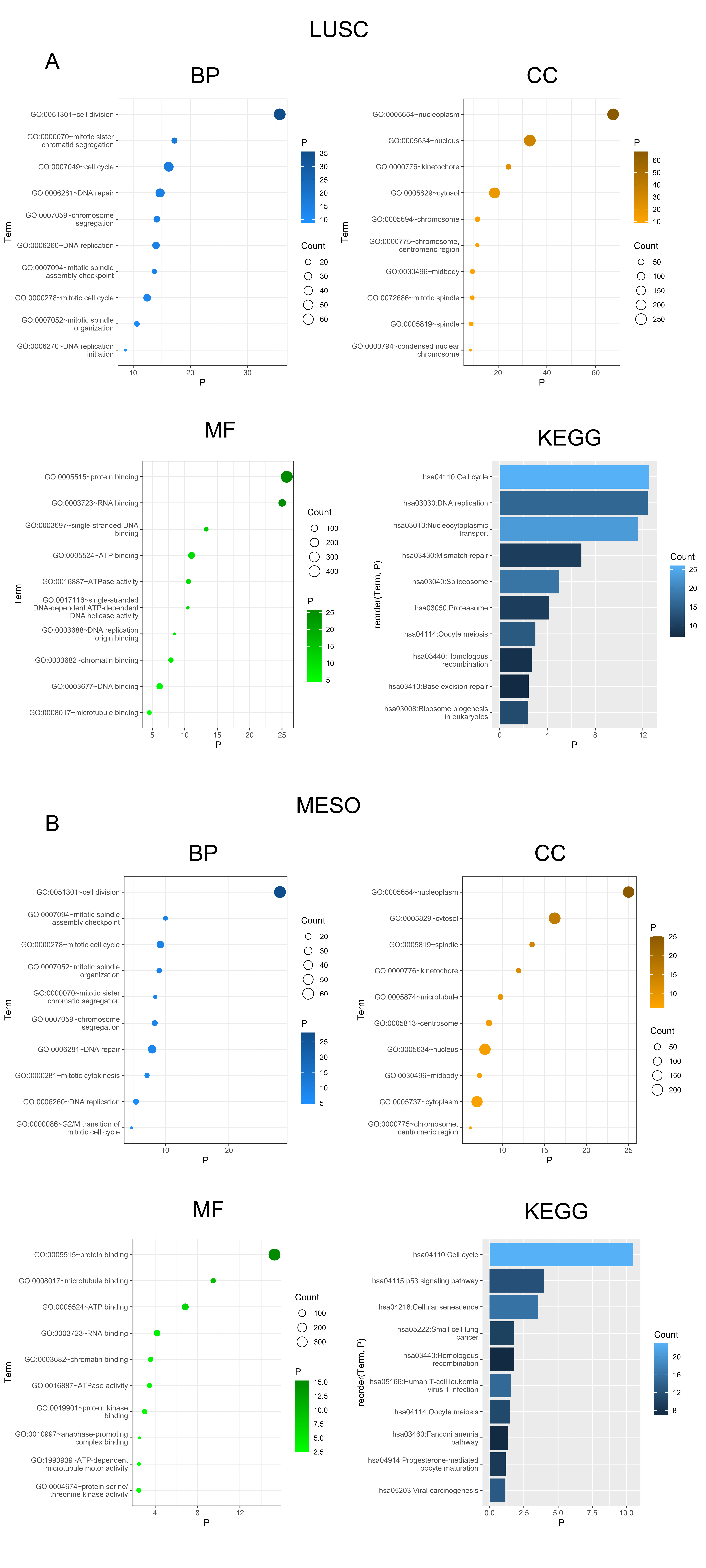

Supplement: Supplementary file 1 [file DataSheet1.zip › Supplementary Figures 1/Figure S11.jpg]

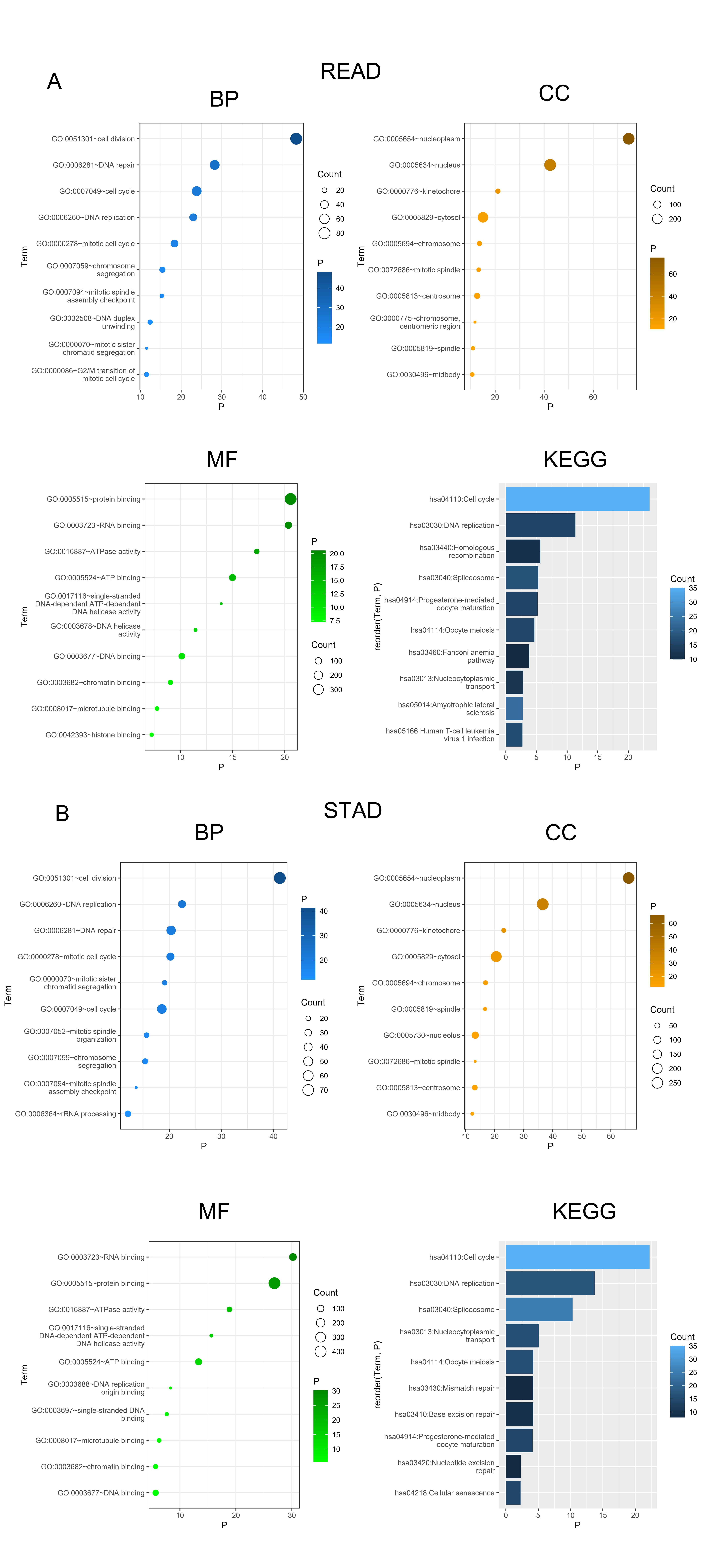

Supplement: Supplementary file 1 [file DataSheet1.zip › Supplementary Figures 1/Figure S12.jpg]

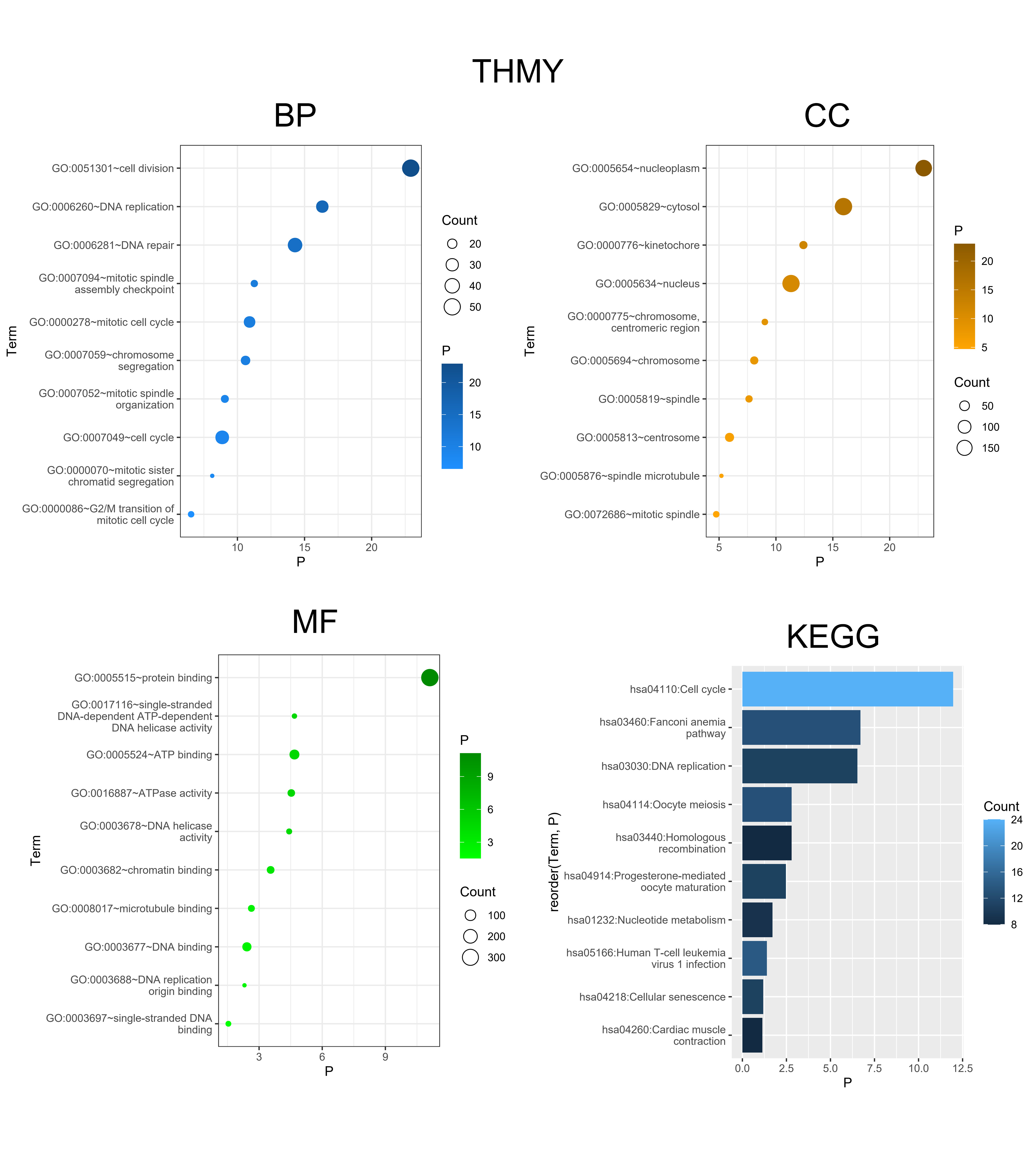

Supplement: Supplementary file 1 [file DataSheet1.zip › Supplementary Figures 1/Figure S13.jpg]

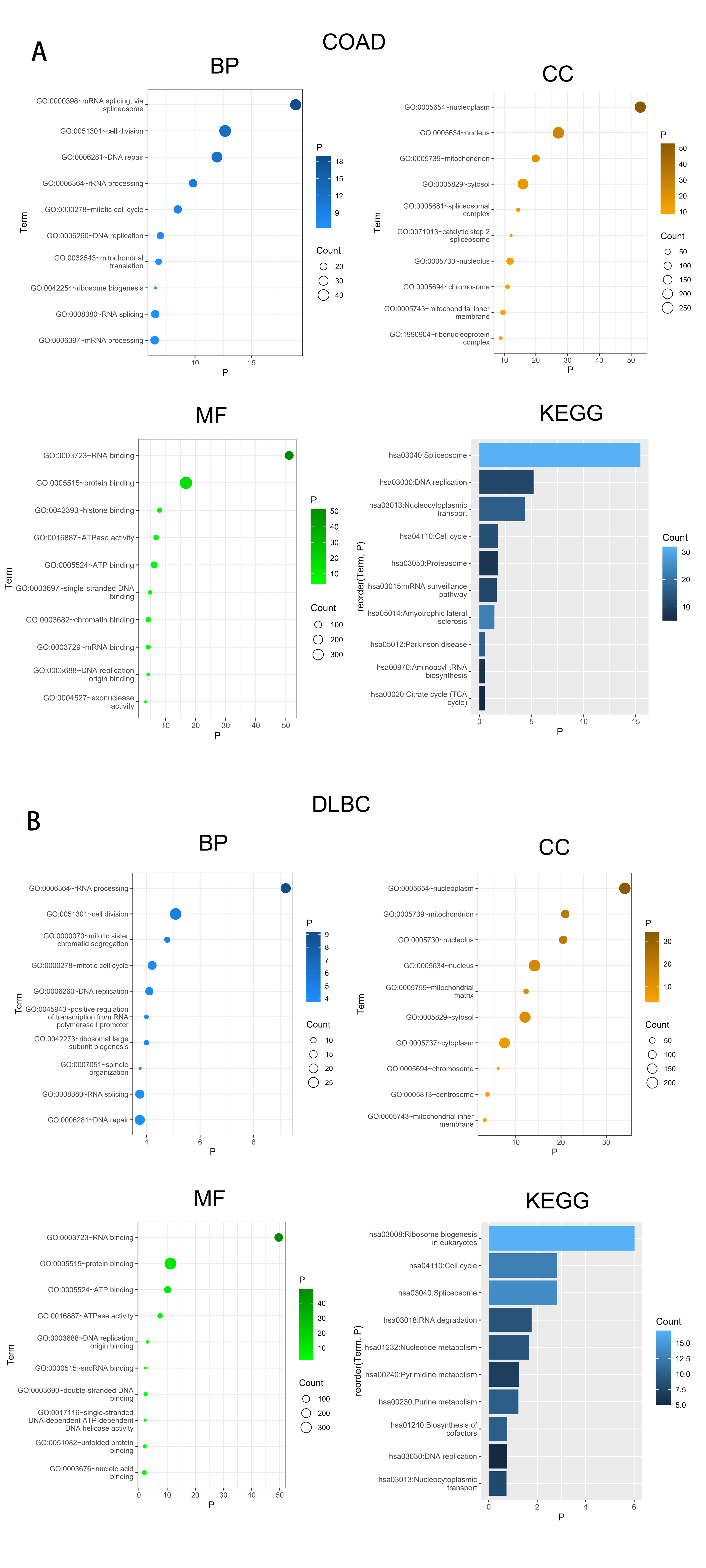

Supplement: Supplementary file 1 [file DataSheet1.zip › Supplementary Figures 1/Figure S7.jpg]

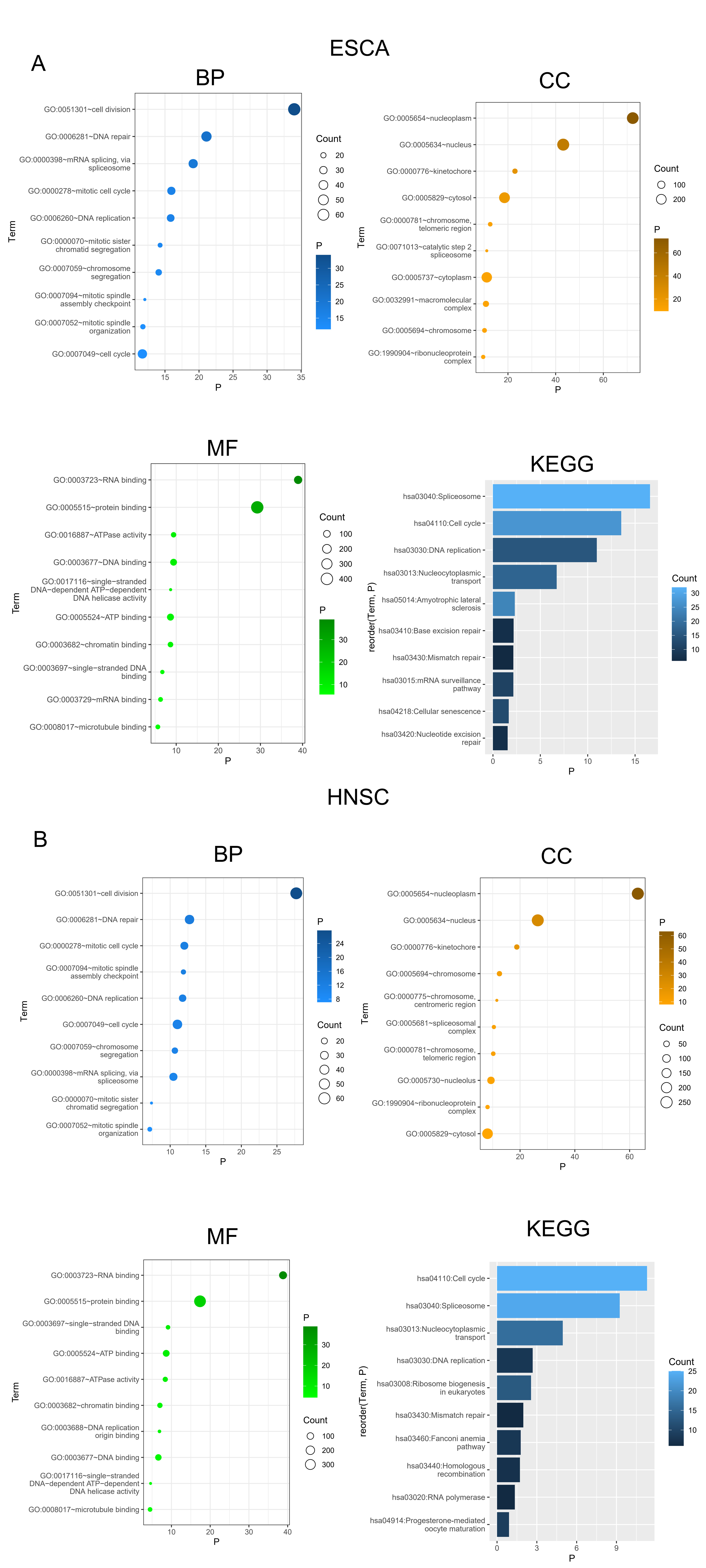

Supplement: Supplementary file 1 [file DataSheet1.zip › Supplementary Figures 1/Figure S8.jpg]

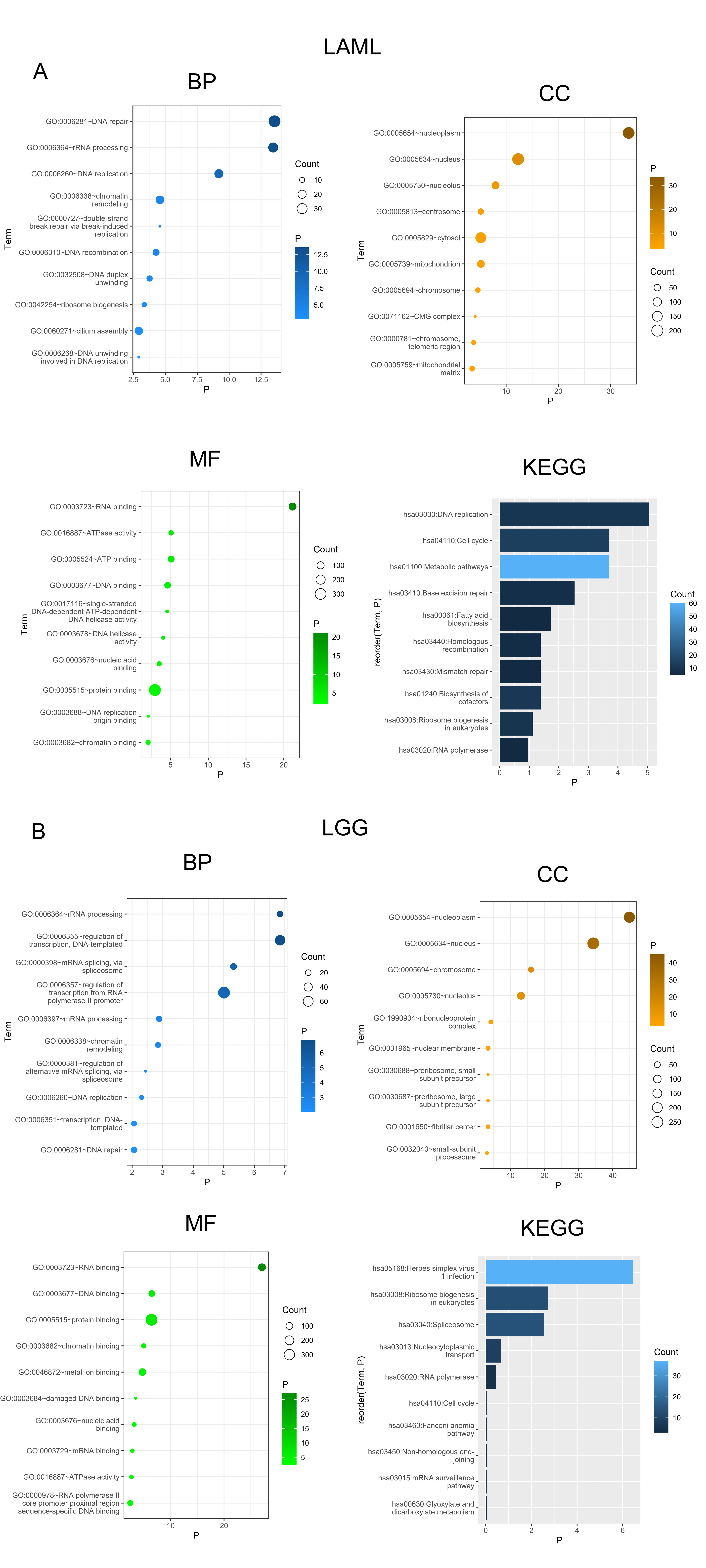

Supplement: Supplementary file 1 [file DataSheet1.zip › Supplementary Figures 1/Figure S9.jpg]

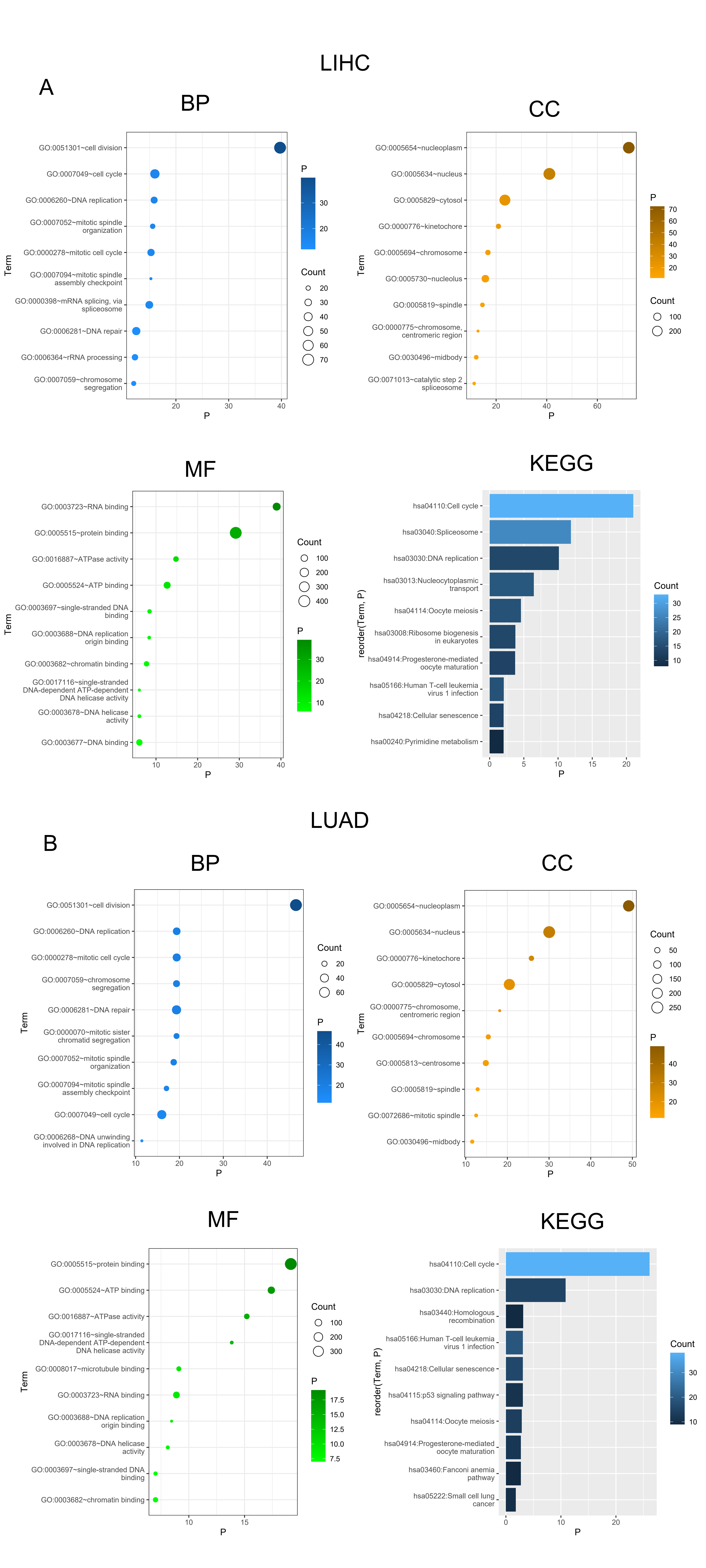

Supplement: Supplementary file 1 [file DataSheet1.zip › Supplementary Figures 1/Figure S10.jpg]

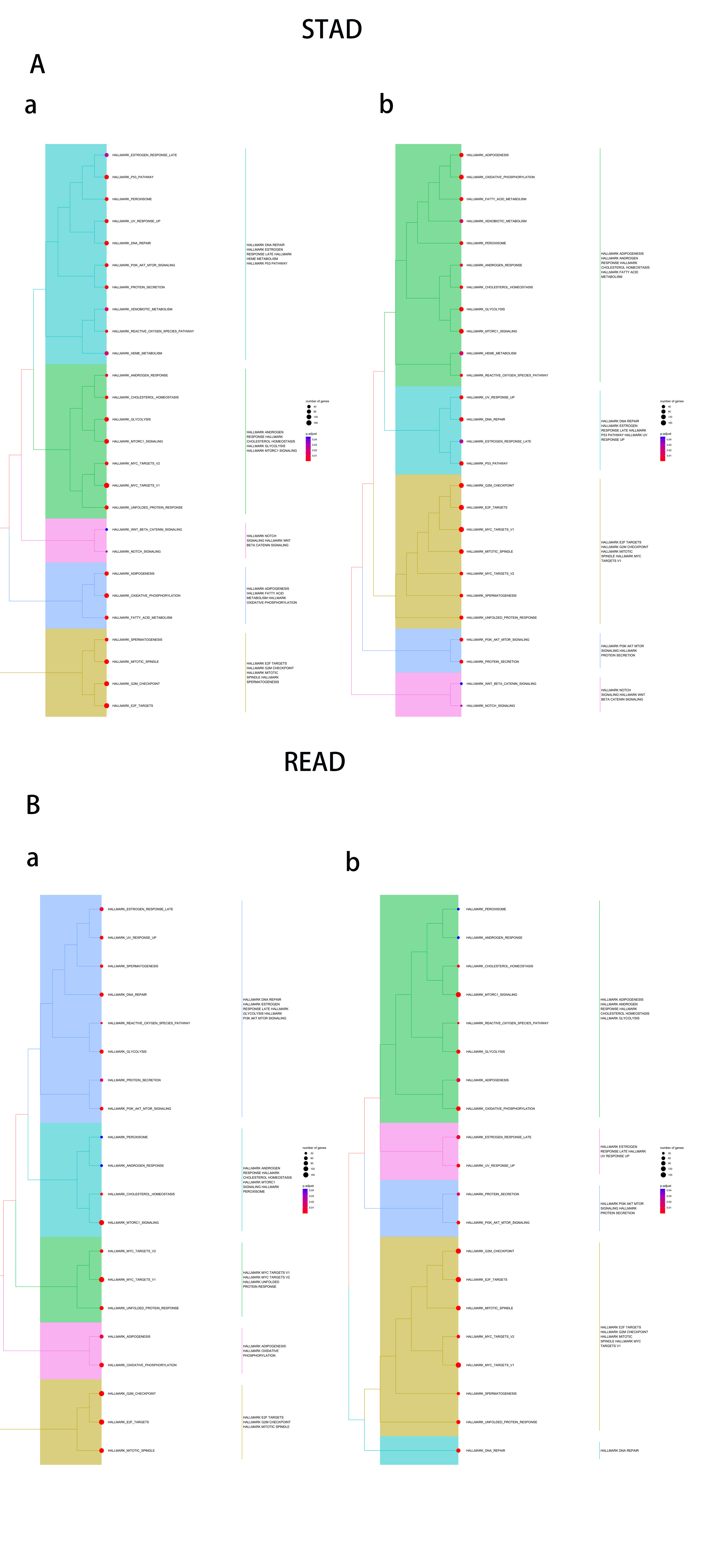

Supplement: Supplementary file 2 [file DataSheet2.zip › Supplementary Figures 2/Figure S20.jpg]

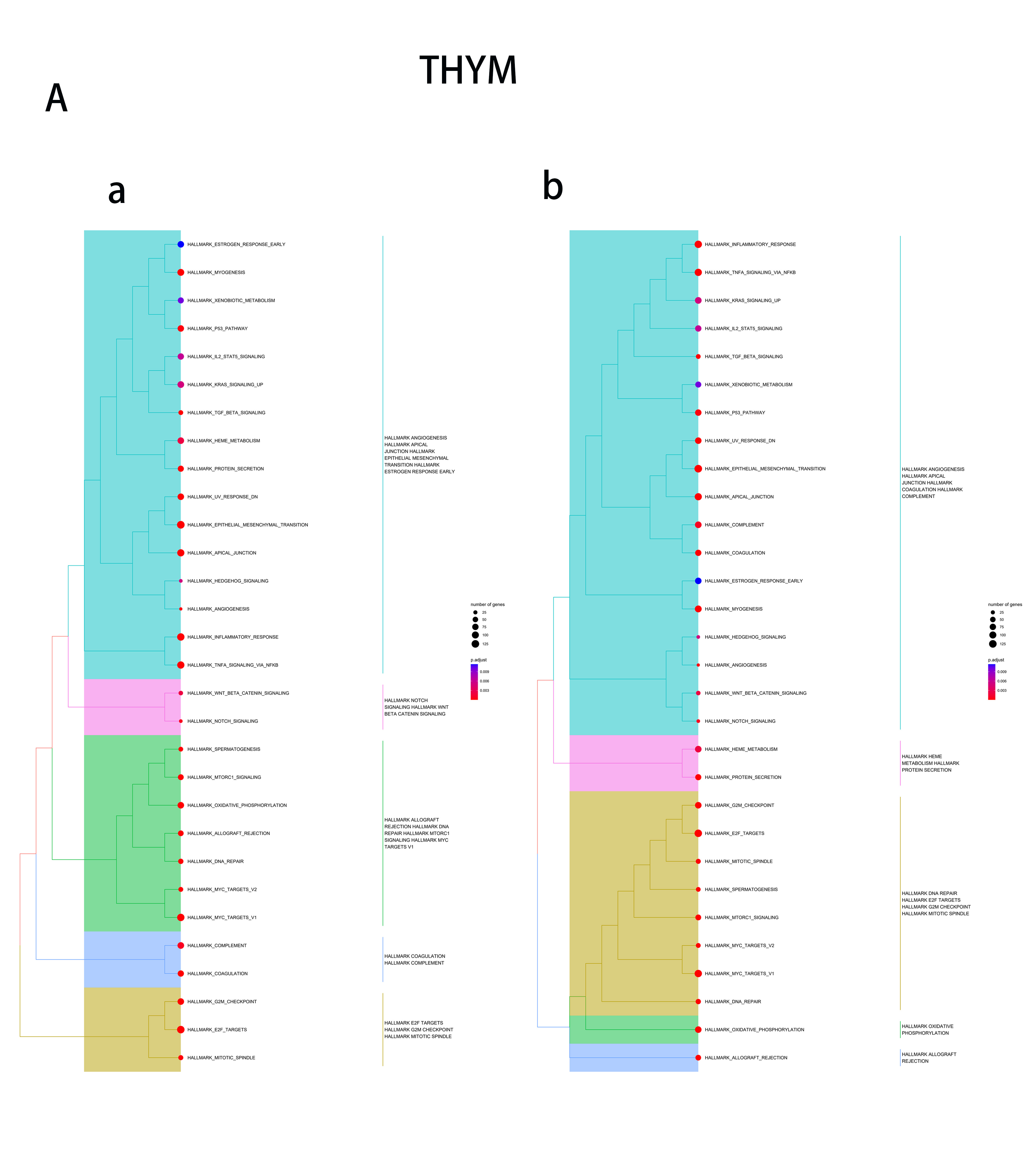

Supplement: Supplementary file 2 [file DataSheet2.zip › Supplementary Figures 2/Figure S21.jpg]

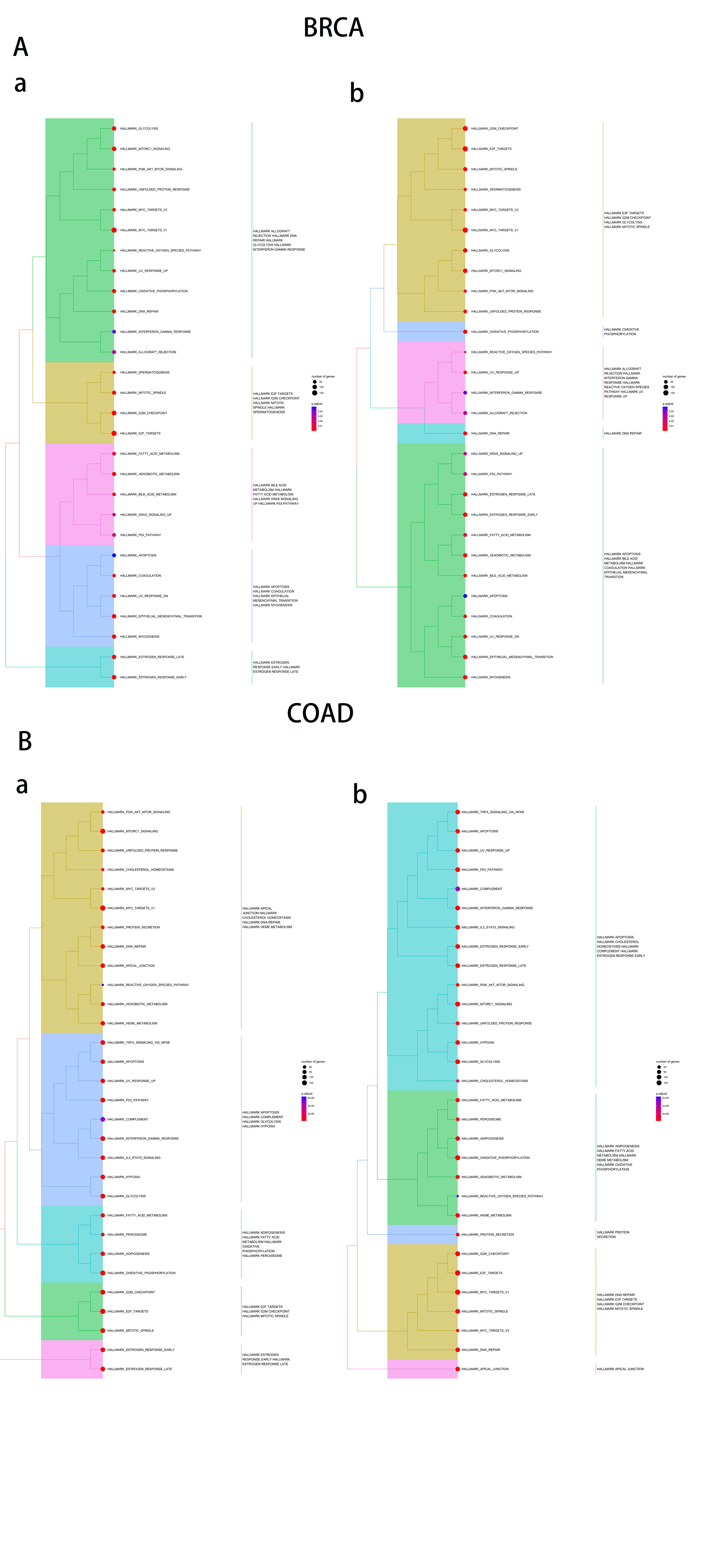

Supplement: Supplementary file 2 [file DataSheet2.zip › Supplementary Figures 2/Figure S14.jpg]

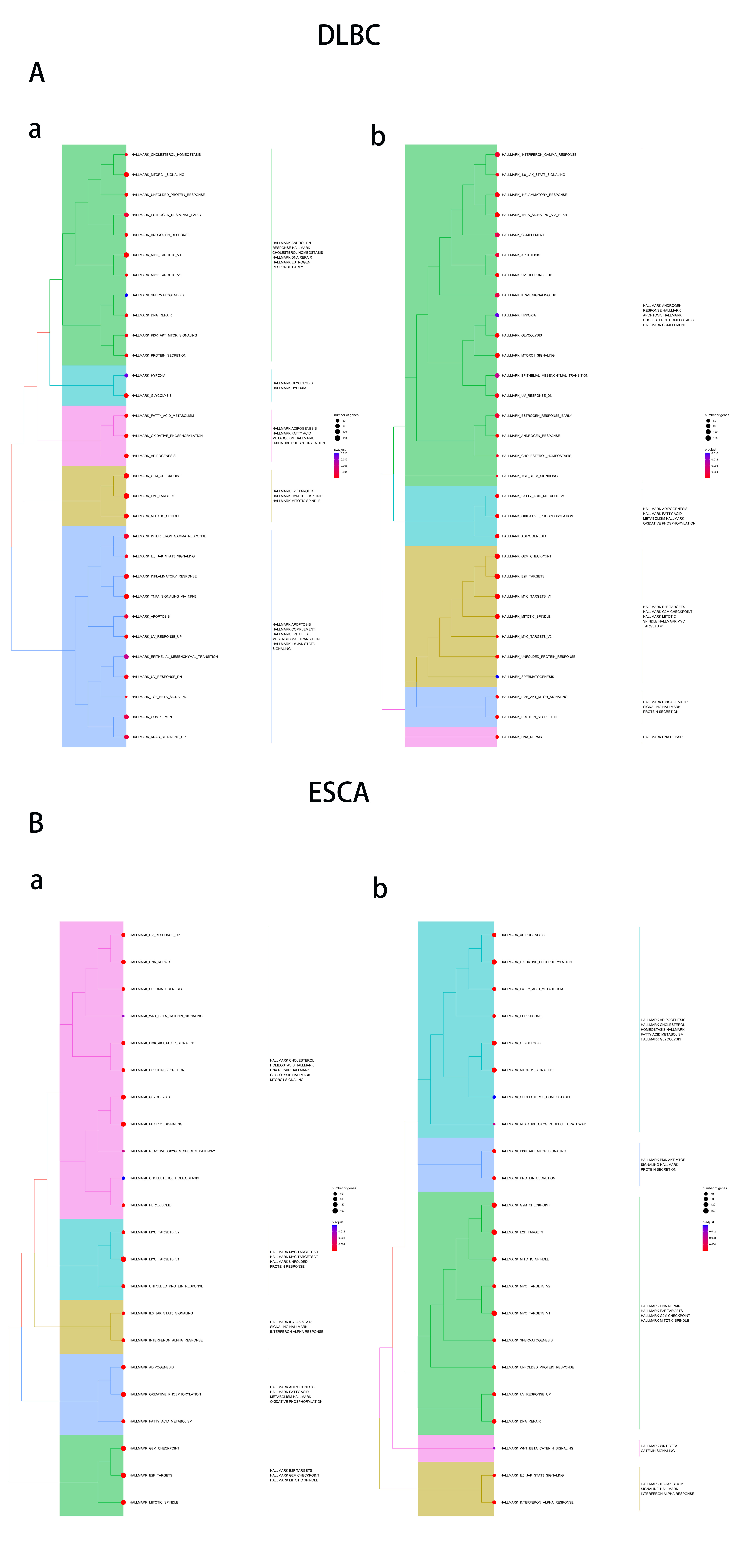

Supplement: Supplementary file 2 [file DataSheet2.zip › Supplementary Figures 2/Figure S15.jpg]

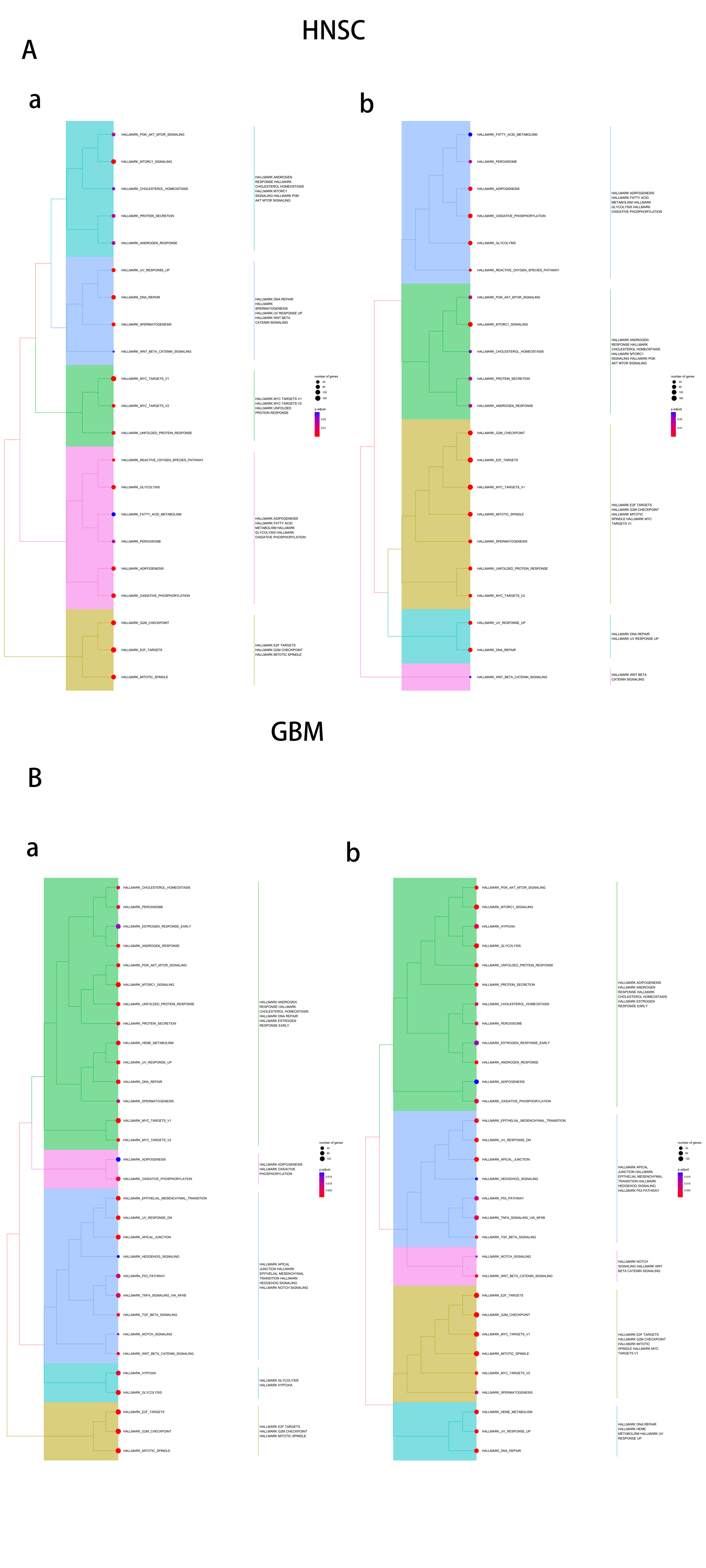

Supplement: Supplementary file 2 [file DataSheet2.zip › Supplementary Figures 2/Figure S16.jpg]

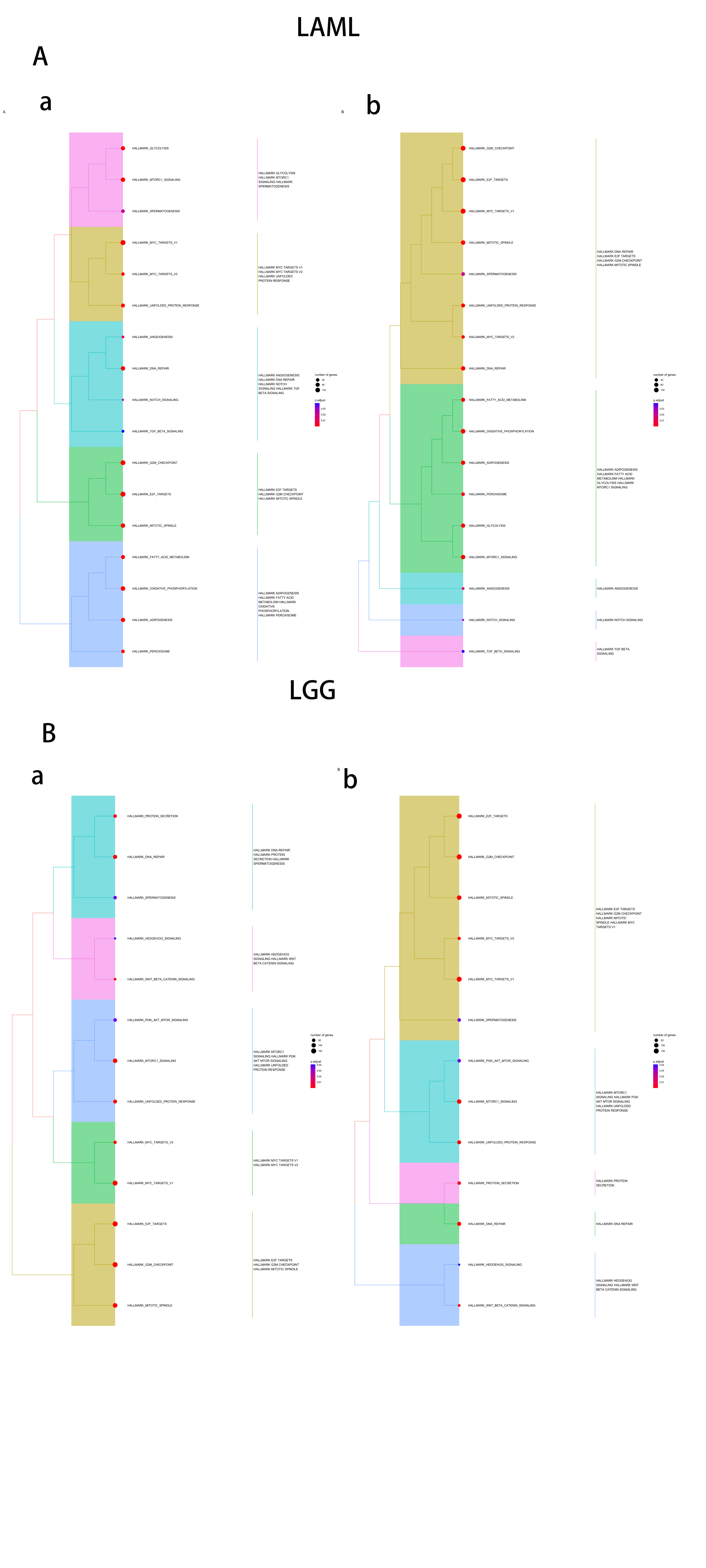

Supplement: Supplementary file 2 [file DataSheet2.zip › Supplementary Figures 2/Figure S17.jpg]

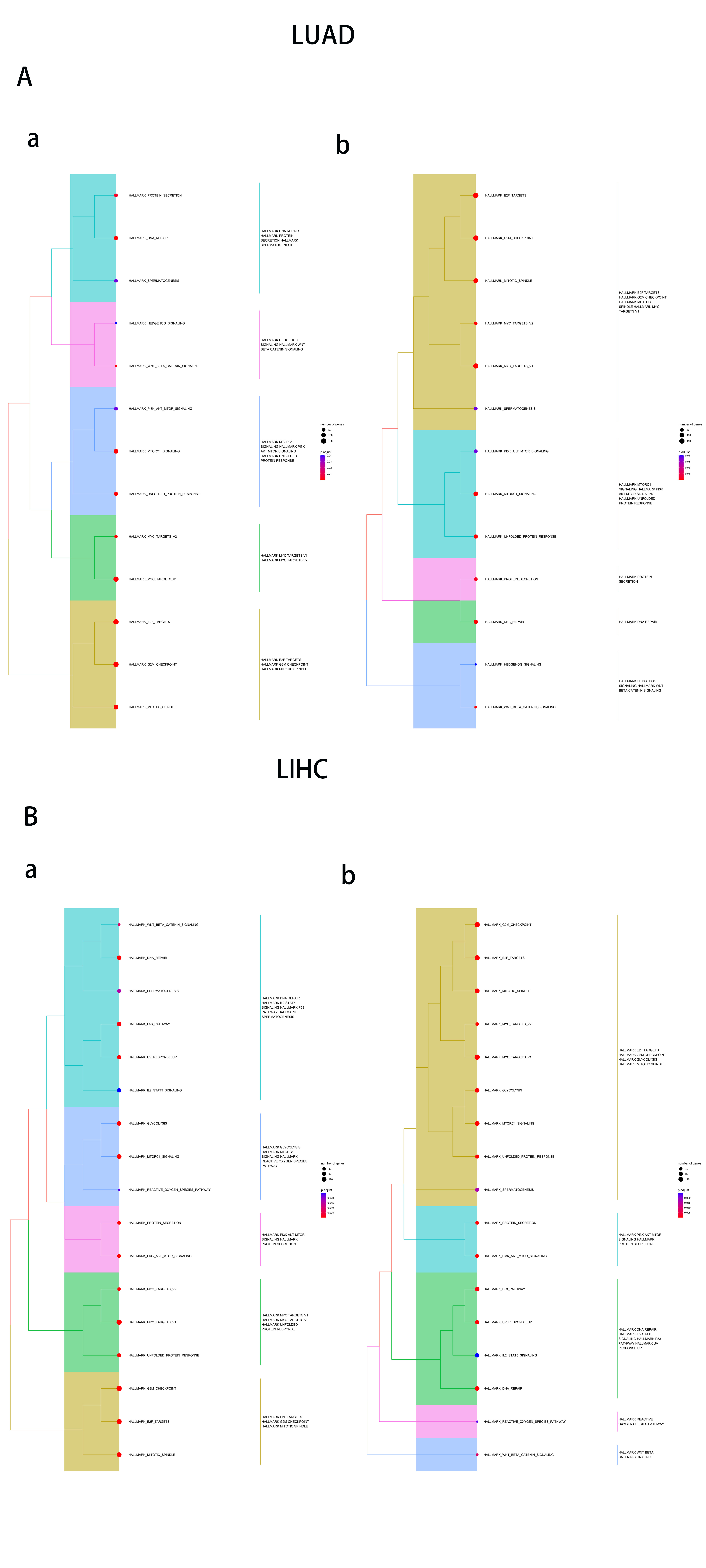

Supplement: Supplementary file 2 [file DataSheet2.zip › Supplementary Figures 2/Figure S18.jpg]

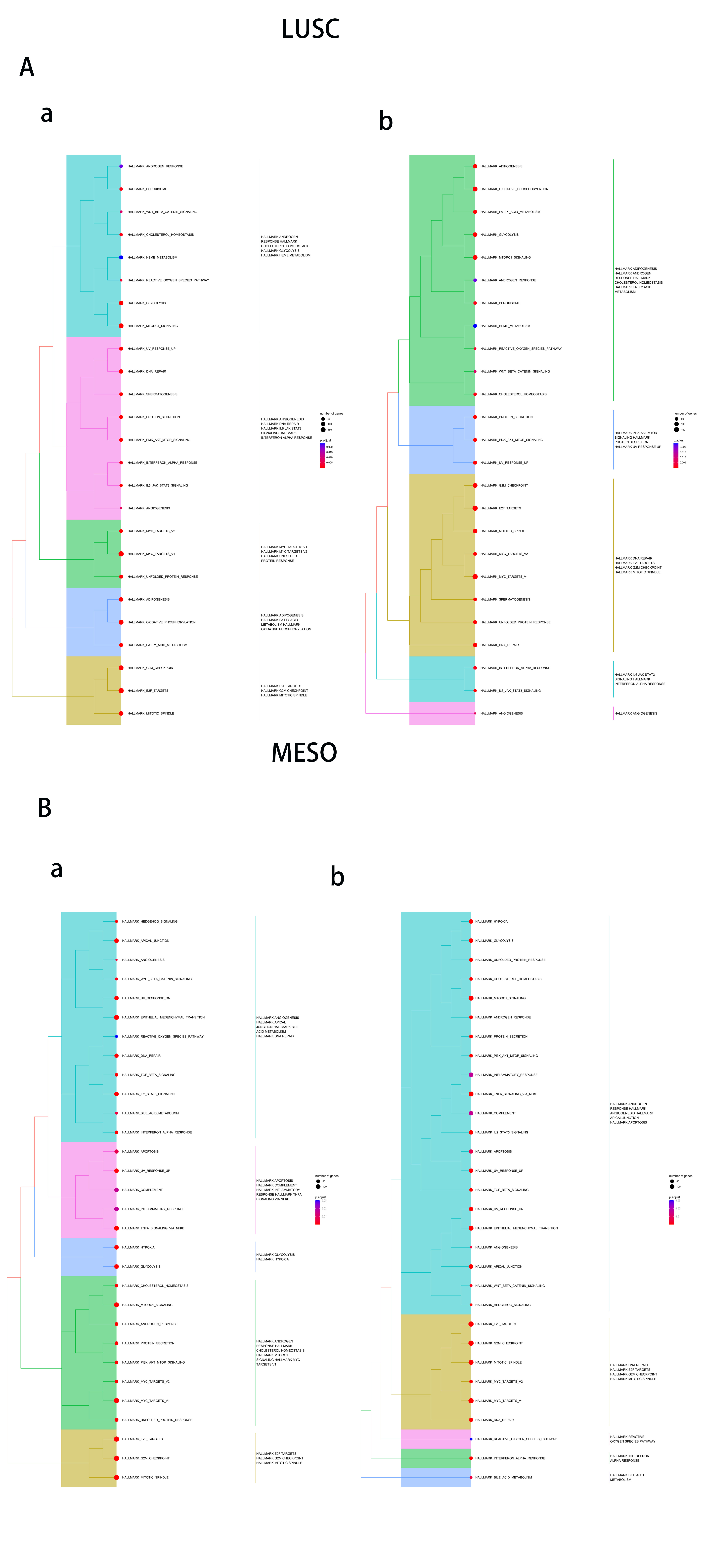

Supplement: Supplementary file 2 [file DataSheet2.zip › Supplementary Figures 2/Figure S19.jpg]
